# Molecular remodeling of cancer-associated fibroblasts in breast cancer patients receiving anti–PD-1 immunotherapy

**DOI:** 10.3389/fonc.2026.1754311

**Published:** 2026-02-24

**Authors:** Khanh Van Do, An Van Tran, Anh Duc Pham, Trang Thu Mac, Thang Luong Pham, Han Ngoc Do

**Affiliations:** 1 Applied Biomedical Research Center, Phenikaa University, Hanoi, Vietnam; 2 BioTuring, San Diego, CA, United States

**Keywords:** anti-PD-1 therapy, breast cancer, CAF subtypes, cancer-associated fibroblasts (CAF), immune checkpoint inhibitors, immune resistance, single-cell RNA sequencing, tumor microenvironment

## Abstract

**Introduction:**

Cancer-associated fibroblasts (CAFs) are integral components of the tumor microenvironment that modulate the response to immune checkpoint inhibitors, particularly in breast cancer. However, the specific roles of CAF subtypes in regulating the efficacy of anti-PD-1 therapy remain poorly elucidated.

**Methods:**

In this study, we reanalyzed single-cell RNA sequencing data from breast cancer patients treated with anti-PD-1 inhibitors to identify CAF subtypes and characterize their molecular signatures. Identified subtypes were further validated using spatial transcriptomics mapping to assess their anatomical niches.

**Results:**

Four distinct CAF subtypes were identified: vascular CAFs (vCAF), myofibroblastic CAFs (myCAF), inflammatory CAFs (iCAF), and antigen-presenting CAF-like (apCAF-like) cells. MyCAFs were localized to fibrotic stromal regions, while iCAFs were found within immune-rich, inflamed areas. In responders, stromal remodeling occurs, characterized by the functional re-education of iCAFs—transitioning to a pro-inflammatory CXCL9-CXCR3 axis—and the concurrent disarmament of vCAF and myCAF populations. Conversely, resistance in non-responders is linked to stromal fortification, driven by the apCAF-like-derived THBS2-CD47 axis and the pathological intensification of the vCAF-derived CXCL12-CXCR4 axis, leading to dysfunctional lymphoid sequestration.

**Discussion:**

Collectively, these findings highlight the critical role of CAF heterogeneity and spatial organization in modulating the response to anti-PD-1 therapy. Targeting subtype-specific stromal modules may represent a promising therapeutic strategy to enhance the efficacy of immunotherapy in breast cancer.

## 1 Introduction

Breast cancer remains one of the leading causes of cancer-related mortality worldwide, and resistance to systemic therapies—including immunotherapy—continues to pose major clinical challenges (1, 2). The tumor microenvironment (TME) plays a crucial role in shaping therapeutic outcomes, with stromal components increasingly recognized as key modulators of treatment response (3). Among these, cancer-associated fibroblasts (CAFs) represent the most abundant stromal cell population and exert profound influence on tumor progression, immune regulation, and drug resistance (4).

Unlike normal fibroblasts, which maintain tissue homeostasis and support wound healing, CAFs adopt tumor-promoting phenotypes that remodel the extracellular matrix (ECM), secrete immunomodulatory cytokines, and orchestrate a pro-tumorigenic microenvironment (5). Advances in single-cell RNA sequencing (scRNA-seq) have revealed that CAFs are not a uniform population but comprise transcriptionally and functionally distinct subtypes (6). These include myofibroblastic CAFs (myCAFs) associated with ECM deposition, inflammatory CAFs (iCAFs) characterized by cytokine signaling, and antigen-presenting CAFs (apCAFs) expressing MHC (Major histocompatibility complex) class II molecules (7). This heterogeneity underlies their diverse and context-dependent effects on tumor immunity.

Despite the transformative potential of immune checkpoint inhibitors (ICIs), such as anti–PD-1 antibodies, only a fraction of breast cancer patients experience durable clinical benefit (8). Emerging evidence indicates that cancer-associated fibroblasts play a critical role in modulating resistance to ICIs. They may contribute to immune evasion by sequestering immune cells from the tumor core or by secreting immunosuppressive factors that inhibit cytotoxic T-cell function (1). However, the precise CAF subtypes involved and the underlying molecular pathways that influence the anti–PD-1 response in breast cancer remain insufficiently understood (7). A deeper understanding of these mechanisms is essential for developing strategies to overcome CAF-mediated resistance and improve ICI efficacy.

To address this gap, we reanalyzed a publicly available single-cell RNA-seq dataset of breast cancer patients treated with anti–PD-1 immunotherapy. Our aim was to systematically define CAF subtypes, delineate their molecular and functional programs, and evaluate their associations with therapeutic response. By focusing on the stromal compartment rather than immune or epithelial cells, this study provides an integrated view of CAF remodeling under checkpoint blockade, highlighting CAF-derived signaling pathways as potential targets to improve immunotherapy efficacy in breast cancer.

## 2 Materials and methods

To investigate stromal heterogeneity in breast cancer, we reanalyzed scRNA-seq data from a publicly available dataset, which includes paired pre-treatment and on-treatment tumor biopsies from patients treated with anti–PD-1 immunotherapy (9). For this study, we focused on 31 treatment-naive patients from the first cohort with operable, non-metastatic breast tumors. The cohort comprised three tumor subtypes—Estrogen receptor-positive (ER+), Human epidermal growth factor receptor 2-positive (HER2+), and Triple-negative breast cancer (TNBC)—and spanned three distinct age groups: young adults, middle-aged adults, and elderly individuals (**Supplementary Figure S1**). Among these patients, 15 harbored ER+ tumors, primarily in middle-aged and elderly adults; 3 had HER2+ tumors, mostly in middle-aged and elderly adults; and the remaining 13 presented with TNBC, distributed across young adults (1 patient), middle-aged adults (5 patients), and elderly adults (7 patients). This distribution highlights the diversity of tumor subtypes and age groups, providing a representative foundation for downstream single-cell analyses.

**Figure 1:**
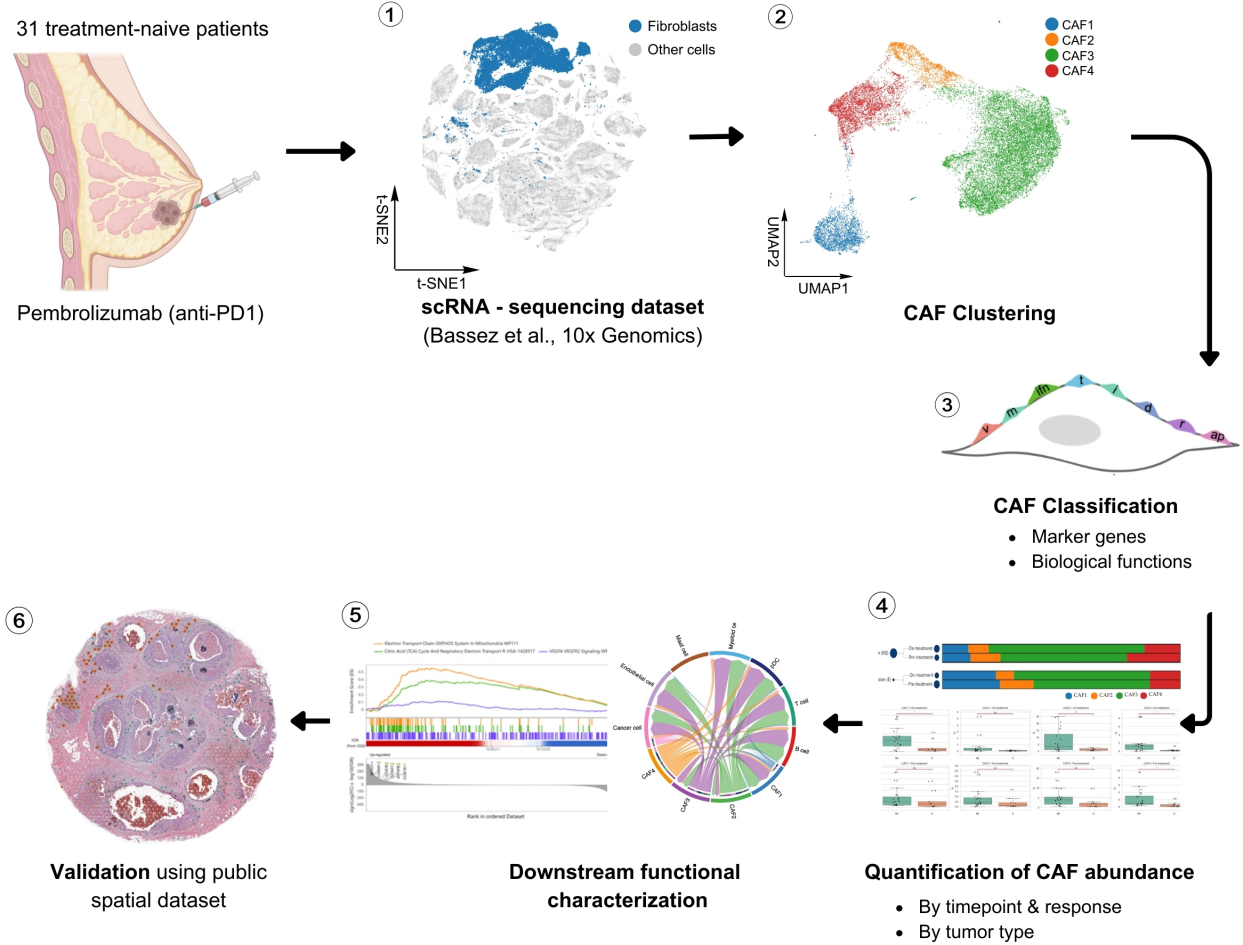
Analytical workflow for CAF-focused reanalysis. The pipeline comprised: (1) preprocessing and stringent QC of scRNA-seq data; (2) identification of transcriptionally distinct CAF clusters via dimensionality reduction and unsupervised clustering; (3) classification of CAF subtypes by differential gene expression and molecular signatures; (4) quantification of CAF subtype abundance across treatment timepoints, response groups, and tumor types; (5) functional characterization through pathway enrichment and cell–cell communication analyses; and (6) anatomical validation of identified CAF niches using public spatial transcriptomics dataset.

The cohort was specifically selected to avoid confounding effects from prior chemotherapy and to ensure the availability of high-quality paired biopsies suitable for single-cell analysis. Each patient received a single dose of pembrolizumab prior to surgery, and tumor tissues were collected both before and shortly after treatment. While the original study primarily emphasized immune cell dynamics and malignant epithelial programs, our analysis concentrated on the stromal compartment, with particular focus on fibroblast populations. The analytical workflow comprised three main steps: first, raw scRNA-seq data were processed and subjected to stringent quality control to ensure reliable resolution at both the cell and gene levels; second, dimensionality reduction and unsupervised clustering were applied to identify transcriptionally distinct fibroblast subsets, which were further characterized via differential expression and pathway enrichment analyses to define molecular programs and subtype-specific signatures; third, the functional relevance and generalizability of the identified CAF subtypes were evaluated through spatial transcriptomics validation (**Figure 1**). All major visualizations, including dimensionality reduction plots, heatmaps, pathway enrichment summaries, and spatial projection figures, were generated and refined using BioVinci (BioTuring Inc.) (10).

### 2.1 Clustering and initial identification of fibroblast subtypes

Fibroblast cell clusters were identified using the Louvain algorithm at a resolution of r = 0.5, implemented through the “Clustering” function of BBrowserX (BioTuring Inc., California, USA) (RRID: SCR 025984) (11), which initially yielded four distinct fibroblast clusters. To capture disease-associated gene expression changes, differential expression (DEG) analysis was performed between relevant cell groups within each cluster. The thresholds applied for DEG analysis included an average log_2_ fold change (log_2_ FC) *>* 0.5 and a false discovery rate (FDR) *<* 0.05, both of which are widely accepted criteria for identifying significantly regulated genes. Volcano plots were generated to visualize these results, enabling rapid assessment of genes that were significantly up- or downregulated and providing a basis for selecting candidate genes implicated in pathogenesis.

To refine classification, marker gene prioritization first incorporated coverage-based metrics—Within-cluster Coverage, Outside-cluster Coverage, and weighted log_2_ FC (Wlog_2_ FC)—to better capture both the prevalence and discriminatory power of candidate genes. We then applied stringent differential marker selection (highlog_2_ FC, low FDR), ranked candidates by specificity and magnitude of upregulation, and assessed their biological relevance. This integrative framework enabled the systematic annotation of CAF clusters and highlighted ambiguous populations requiring cautious interpretation.

Each fibroblast cluster was further characterized by applying the “Marker Genes” function, which facilitated the assignment of putative CAF subtypes through comparison with established or novel cell-type markers. To confirm the robustness and specificity of these assignments, fibroblasts were separated from other cell types using the “Sub-Cluster” function. Enrichment analyses were subsequently performed on the DEG sets of each fibroblast cluster under both conditions (NC – Normal Control, BC – Breast Cancer) to refine subtype classification.

### 2.2 Downstream functional and interaction characterization of CAF subtypes

After defining fibroblast clusters and assigning putative subtype identities, we next performed molecular and functional profiling to delineate their biological programs and potential interactions within the tumor microenvironment. Two complementary analyses were conducted: pathway enrichment analysis of subtype- specific DEGs and inference of intercellular communication networks.

For pathway enrichment analysis, DEGs of each CAF cluster were submitted to the Enrichr platform (RRID: SCR 001575) (12), with functional annotations derived from multiple curated resources including Reactome (RRID: SCR 003485) (13), Wikipathways (RRID: SCR 002134) (14), and the Gene Ontology (GO) biological processes database (RRID: SCR 002811) (15). Significance was assessed using adjusted p-values with FDR *<* 0.05. This approach enabled the systematic identification of pathways enriched in each CAF subtype, providing insight into distinct transcriptional programs underlying extracellular matrix remodeling, angiogenesis, metabolism, protein synthesis, and immune modulation.

To evaluate potential cell–cell interactions, ligand–receptor pairing was analyzed using CellPhoneDB (v2.1.7) (RRID: SCR 017054) (16) and cross-validated with BBrowserX’s built-in cell-cell communication inference tool. Only statistically significant interactions (p-values *<* 0.05, permutation test) were retained. We specifically examined pathways with known relevance to tumor-immune crosstalk. Interaction networks were visualized to highlight both outgoing (CAF-derived ligands) and incoming (CAF-expressed receptors) signaling axes, enabling comparative mapping across CAF subtypes. This integrated molecular and functional profiling strategy provided the foundation for subsequent interpretation of CAF subtype-specific roles in shaping the tumor microenvironment and modulating responses to immune checkpoint blockade.

### 2.3 Data validation by comparison with previous studies

To examine the spatial organization of CAF programs identified in our scRNA-seq analysis, we analyzed a publicly available breast cancer spatial transcriptomics dataset generated using the 10x Genomics Visium FFPE platform (2021) (RRID: SCR 023571) (17). The dataset was accessed via Talk2Data (18) and processed using the SpatialX platform (BioTuring Inc.) (19). The analyzed tissue corresponds to an FFPE section (Block 738811QB, Section 1) from a grade II breast carcinoma of a 73-year-old Asian female, encompassing regions of ductal carcinoma *in situ* and invasive carcinoma. As no information on treatment status or clinical response was available, the analysis was restricted to baseline spatial organization. Spatial spots were clustered using the Louvain algorithm (resolution = 5). CAF subtypes were assigned based on marker gene signatures derived from our scRNA-seq analysis and projected onto tissue coordinates to assess their spatial distribution.

## 3 Results

### 3.1 CAF subtypes with distinct roles in the tumor microenvironment: vCAF, myCAF, iCAF, and apCAF-like phenotypes

To investigate the functional heterogeneity of CAFs within the tumor microenvironment, we identified four distinct CAF subtypes based on their gene expression profiles: CAF1 as vascular CAFs (vCAF), CAF2 as myofibroblastic CAFs (myCAF), CAF3 as inflammatory CAFs (iCAF), and CAF4 as antigen-presenting CAF-like (apCAF-like) CAFs. Each subtype exhibited unique molecular signatures and pathway enrichments, suggesting distinct roles in tumor progression and therapy resistance.

**Figure 2:**
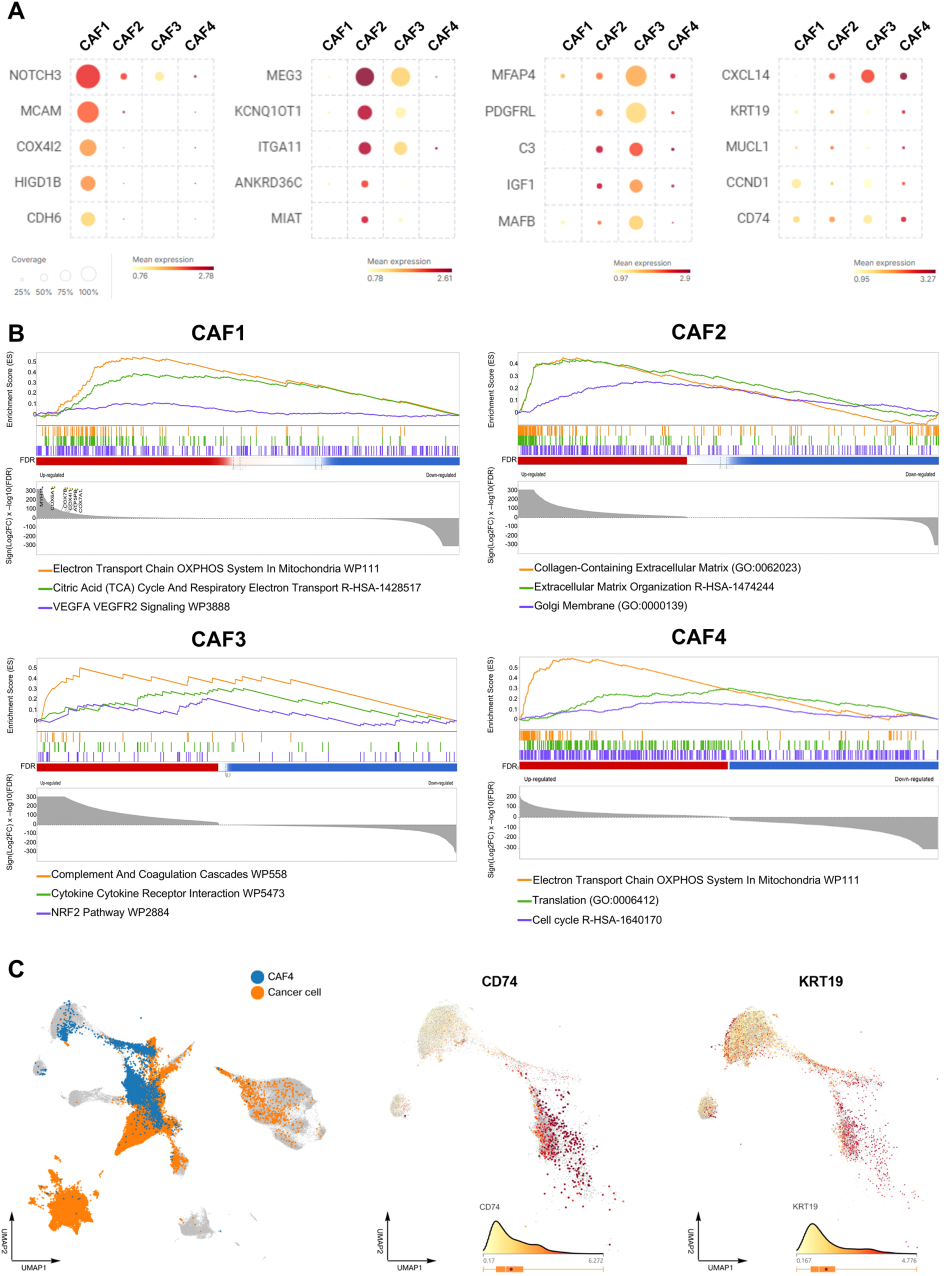
Functional characterization of CAF subtypes in breast cancer and specific features of CAF4. **(A)** Heatmap of the top 5 marker genes across the four CAF subtypes, selected based on F1 score and Within-cluster Coverage. Gene expression is shown on an absolute scale, excluding non-expressed cells. For each gene, mean expression and coverage (percentage of expressing cells) are shown below the heatmap. **(B)** Pathway enrichment analysis of CAF subtypes. CAF1 is enriched in VEGFA-VEGFR2 signaling, Rho GTPase pathways, and mitochondrial respiration. CAF3 shows enrichment in Complement and Coagulation Cascades, Cytokine-Cytokine Receptor Interaction, and the NRF2 (Nuclear factor erythroid 2-related factor 2) pathway. CAF4 is enriched in pathways related to metabolic activation and proliferation. **(C)** UMAP embedding of malignant cells and CAF subtypes showing spatial overlap between CAF4 and epithelial cancer cells. Feature plots of *CD74* (apCAF marker) and *KRT19* (epithelial marker) highlight CAF4’s hybrid signature compared to other CAF populations.

CAF1 exhibited a robust gene expression profile associated with vascular functions, with key markers including Notch Receptor 3 (*NOTCH3*), Melanoma Cell Adhesion Molecule (*MCAM*), Cytochrome C Oxidase Subunit 4I2 (*COX4L2*), HIG1 Hypoxia Inducible Domain Family Member 1B (*HIGD1B*), and Cadherin 6 (*CDH6*) (**Figure 2A**). *NOTCH3*, involved in angiogenesis and endothelial cell signaling, reinforced the vascular phenotype of CAF1 (20). *MCAM* and *CDH6*, adhesion molecules critical for cell-cell interactions, suggest CAF1’s role in vessel formation and stabilization (21). The NOTCH Regulated Ankyrin Repeat Protein (*NRARP*) gene, regulating NOTCH signaling, further supports the angiogenic profile (**Supplementary Figure S2A**; **Supplementary Data 2**) (22). Additionally, the expression of Gap Junction Protein Alpha 4 (*GJA4*) and G Protein-Coupled Receptor 4 (*GPR4*), along with Potassium Voltage-Gated Channel Subfamily A Member 5 (*KCNA5*), RAS Like Glutamate Rich (*RERGL*), and Calsequestrin 2 (*CASQ2*), highlights CAF1’s potential role in modulating endothelial function and vascular responses to tumor growth (23–25). Pathway analysis further enriched CAF1 in mitochondrial energy metabolism pathways, including the Electron Transport Chain, Mitochondrial ATP Synthesis, and the TCA cycle (Citric Acid cycle), underscoring its energetic support for tumor progression. This, combined with pathways related to RNA processing, splicing, and VEGFA–VEGFR2 signaling, indicates CAF1’s active involvement in angiogenesis, vessel stabilization, and possibly immune exclusion via vascular-mediated mechanisms (**Figure 2B**; **Supplementary Data 3**).

Exhibiting a transcriptional signature indicative of myofibroblastic differentiation and ECM remodeling, CAF2 expresses key markers such as Maternally Expressed 3 (*MEG3*), KCNQ1 Opposite Strand/Antisense Transcript 1 (*KCNQ1OT1*), Integrin Subunit Alpha 11 (*ITGA11*), Ankyrin Repeat Domain 36C (*ANKRD36C*), Myocardial Infarction Associated Transcript (*MIAT*), and ADAM Metallopeptidase With Thrombospondin Type 1 Motif 6 (*ADAMTS6*) (**Figure 2A**). *ITGA11*, an integrin involved in ECM attachment and fibroblast migration, highlights CAF2’s role in promoting tissue stiffness (26). The long noncoding RNAs Nuclear Paraspeckle Assembly Transcript 1 (*NEAT1*), *MIAT*, Xist Ribonucleoprotein (*XIST*), and *KCNQ1OT1* suggest transcriptional reprogramming, typical of fibroblast activation and fibrosis (**Supplementary Figure S2B**; **Supplementary Data 2**) (27). *ADAMTS6*, a metalloproteinase, plays a critical role in ECM turnover, reinforcing CAF2’s function in ECM remodeling (28). Pathway analysis reveals strong enrichment of ECM-related pathways, such as Collagen-Containing Extracellular Matrix, Extracellular Matrix Organization, and Collagen Formation, confirming CAF2’s role in desmoplasia, mechanotransduction, and stromal stiffening (**Figure 2B**; **Supplementary Data 3**).

A gene signature enriched in inflammatory and immunomodulatory pathways characterizes CAF3, with key markers such as Microfibril Associated Protein 4 (*MFAP4*), Platelet Derived Growth Factor Receptor Like (*PDGFRL*), Complement C3 (*C3*), Insulin Like Growth Factor 1 (*IGF1*), and MAF BZIP Transcription Factor B (*MAFB*) (**Figure 2A**). *C3*, a complement system component, indicates CAF3’s role in immune modulation through inflammation and immune cell recruitment (29). *PDGFRL*, a receptor involved in stromal-immune interactions, and *IGF1*, promoting tumor survival, further support this function (30, 31). Pathway analysis revealed a significant intensification of secretory programs within this subtype (**Figure 2B**, **Supplementary Data 3**). The Complement and Coagulation Cascades (WP558) pathway, featuring *SERPING1*, *C1S*, *C1R*, and Complement Factor D (*CFD*), establishes iCAFs as a primary source of innate immune modulators. Concurrently, the Cytokine-Cytokine Receptor Interaction (WP5473) axis, involving *IL6ST*, *CXCL12*, *CXCL14*, and *CCL2*, positions iCAFs as a central signaling hub. Additionally, iCAFs exhibit high metabolic plasticity via the NRF2 Pathway (WP2884), characterized by antioxidant genes such as Superoxide Dismutase 3 (*SOD3*), Glutathione Peroxidase 3 (*GPX3*), and Hepatocyte Growth Factor (*HGF*). Beyond immunomodulation, pro-angiogenic factors like Retinoic Acid Receptor Responder 1 (*RARRES1*) and Vascular Endothelial Growth Factor D (*VEGFD*) suggest that iCAFs foster metastasis through vascular remodeling (**Supplementary Figure S2C**; **Supplementary Data 2**) (32, 33). These findings establish iCAFs as essential orchestrators of an inflammatory, tumor-supportive stroma.

CAF4, designated as an apCAF-like subtype, exhibited a mosaic transcriptional signature with features of stromal, epithelial, and immune-related cells. Key markers such as C-X-C Motif Chemokine Ligand 14 (*CXCL14*), Keratin 19 (*KRT19*), Mucin Like 1 (*MUCL1*), CD74 Molecule (*CD74*), and Major Histocompatibility Complex, Class II, DP Alpha 1 (*HLA-DPA1*) suggest a hybrid phenotype, with *CXCL14* involved in immune cell recruitment and *KRT19* marking epithelial-like features (**Figure 2A**). *CD74*, an antigen-presenting molecule, supports CAF4’s potential role in immune modulation, similar to that of antigen-presenting CAFs (apCAF) (34). To ensure cellular identity and exclude potential doublets or technical artifacts, we performed stringent quality control and expression profiling. CAF4 cells consistently displayed gene counts within a normal range (Number of genes *<* 3,000), arguing against technical artifacts combining multiple cell types (**Supplementary Figure S2E**). Furthermore, while low-level expression of epithelial-associated markers and other transcripts such as *HLA-DPA1*, *MUCL1*, and Trefoil Factor 3 (*TFF3*) were detectable (**Supplementary Figure S2D**; **Supplementary Data 2**) (35, 36), CAF4 cells maintained high expression of core fibroblast markers, including Collagen Type I Alpha 1 Chain (*COL1A1*), Collagen Type III Alpha 1 Chain (*COL3A1*), Decorin (*DCN*), and Lumican (*LUM*), confirming their lineage as *bona fide* fibroblasts (**Supplementary Figure S2F**). These findings, distinct clustering observed in UMAP embedding, confirm that CAF4 represents a specialized fibroblast state adapted to the immune-rich niche rather than epithelial contamination (**Figure 2C**) (37). Pathway analysis further reveals that CAF4 is enriched in mitochondrial energy metabolism (e.g., Electron Transport Chain and Mitochondrial ATP Synthesis) and translation-related programs (e.g., Cap-Dependent Translation Initiation and Ribosomal Subunit Joining) (**Figure 2B**), indicating elevated energetic and biosynthetic activity. Importantly, CAF4 also shows significant enrichment of antigen processing and presentation pathways, including cross-presentation of exogenous antigens via endosomal compartments and MHC class I–mediated antigen presentation, consistent with an apCAF-like functional phenotype (**Supplementary Data 3**).

### 3.2 Modelling of CAF subpopulations heterogeneity in breast cancer

**Figure 3:**
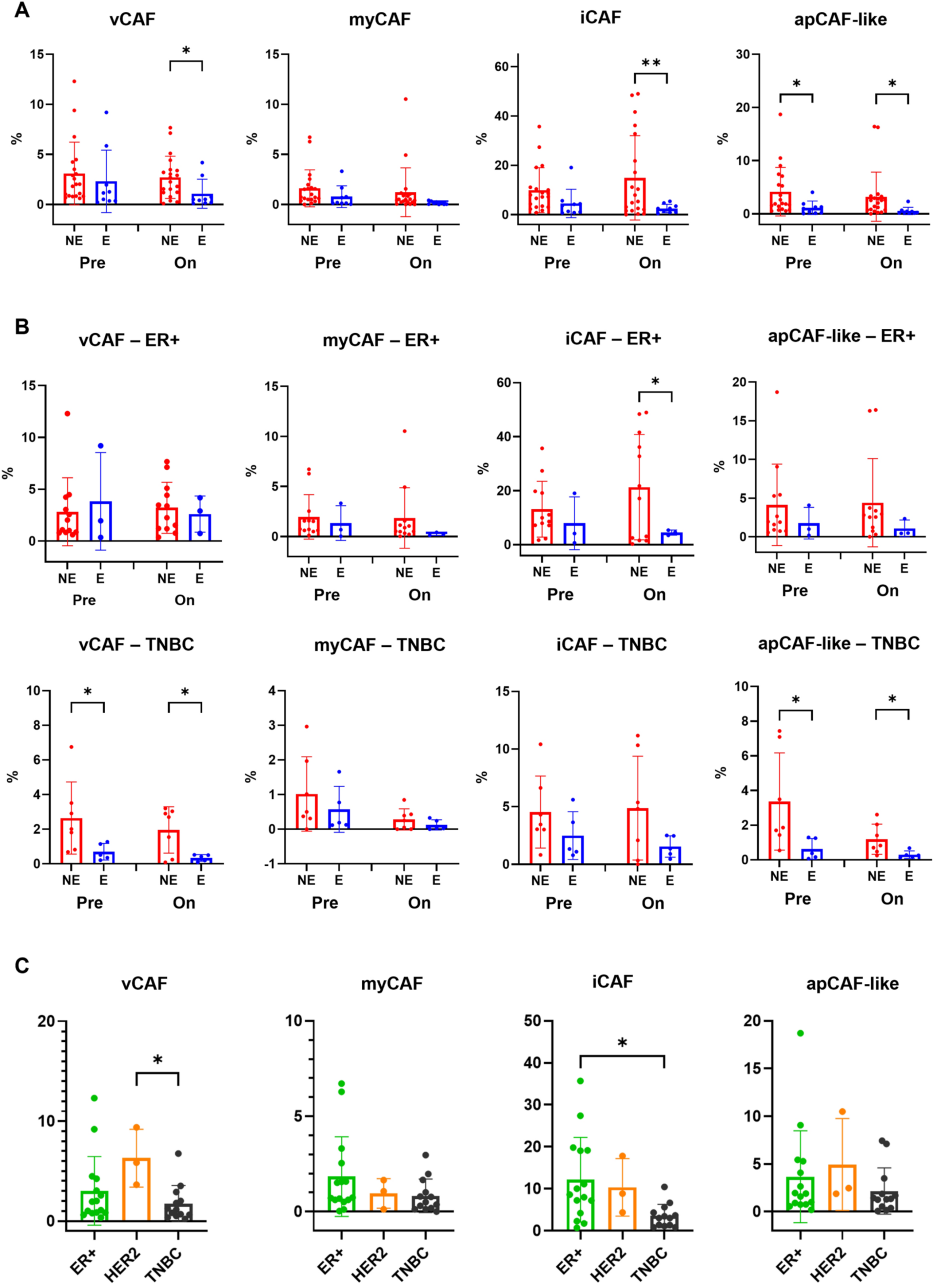
Analysis of CAF subsets in breast cancer patients. **(A)** Proportions of CAF subtypes in breast cancer patients before treatment (Pre) and during treatment (On), stratified by response (E) and non-response (NE). **(B)** Proportions of CAF subtypes in ER+ and TNBC patients before and during treatment, stratified by E and NE. **(C)** Comparison of CAF subtype proportions among breast cancer subtypes (TNBC, HER2+, and ER+) before treatment. In **(A)** and **(B)**, statistical significance was assessed using the unpaired t-test with Welch’s correction. In **(C)**, statistical significance was assessed using one-way ANOVA followed by Tukey’s HSD *post hoc* test. Exact P values are shown in the plots. ∗*P <* 0.05, ∗∗*P <* 0.01. Data are presented as boxplots with individual values overlaid; boxes represent the median and interquartile range, and whiskers denote minimum and maximum values. All analyses were performed using GraphPad Prism v10.5 (RRID: SCR 002798).

We assessed the association between the characterized CAF subtypes and the anti-PD-1 response (using Welch’s t-test) (**Figure 3A**). The vCAF, iCAF, and apCAF-like subtypes were significantly enriched in non-responders compared to responders during treatment. In contrast, the myCAF subtype remained at low, unchanged levels. At the pre-treatment baseline, only the apCAF-like subtype showed modest enrichment in non-responders, suggesting a potential baseline predictive value. Upon treatment, both the vCAF and iCAF subtypes were elevated in non-responders, reflecting their dynamic roles in sustaining immunosuppressive signaling and driving adaptive therapy resistance. The apCAF-like subtype remained higher in non-responders at both pre-treatment and on-treatment stages, reinforcing its stable contribution to a resistant stromal niche. Conversely, responders consistently showed low levels of these three CAF subtypes, indicating a less suppressive stromal environment that may facilitate T cell infiltration and anti-tumor immunity. Collectively, these results suggest that the apCAF-like/admixture subtype serves as a baseline predictor, while the vCAF and iCAF subtypes are numerically associated with adaptive resistance; the myCAF subtype appears to have minimal impact on the treatment outcome.

To explore context-specific roles, we examined the vCAF, iCAF, and apCAF-like subtypes within different breast cancer types (**Figure 3B**). The vCAF and apCAF-like subtypes were found to be enriched in TNBC. Crucially, the iCAF subtype showed a selective increase in ER+ tumors during treatment, highlighting a differential response mechanism. HER2+ samples were excluded from comprehensive statistical analysis due to limited sample size (*n* = 3). However, preliminary cross-subtype comparisons (**Figure 3C**) indicated that the vCAF subtype appeared higher in HER2+ compared to TNBC, and the iCAF subtype was significantly enriched in ER+ relative to TNBC. The myCAF and apCAF-like subtypes showed no significant differences across these breast cancer subtypes. These findings underscore the heterogeneity of CAF distribution and function across breast cancer subtypes, confirming the apCAF-like subtype as a baseline predictor and the vCAF and iCAF subtypes as adaptive drivers of resistance, with the iCAF subtype showing a distinct, selective enrichment in ER+ tumors during therapy. The observations related to HER2+ tumors remain inconclusive due to sample limitations.

### 3.3 Anti-PD-1 therapy orchestrates dual stromal reprogramming: re-education of iCAFs and functional disarmament of vCAFs and myCAFs

**Figure 4:**
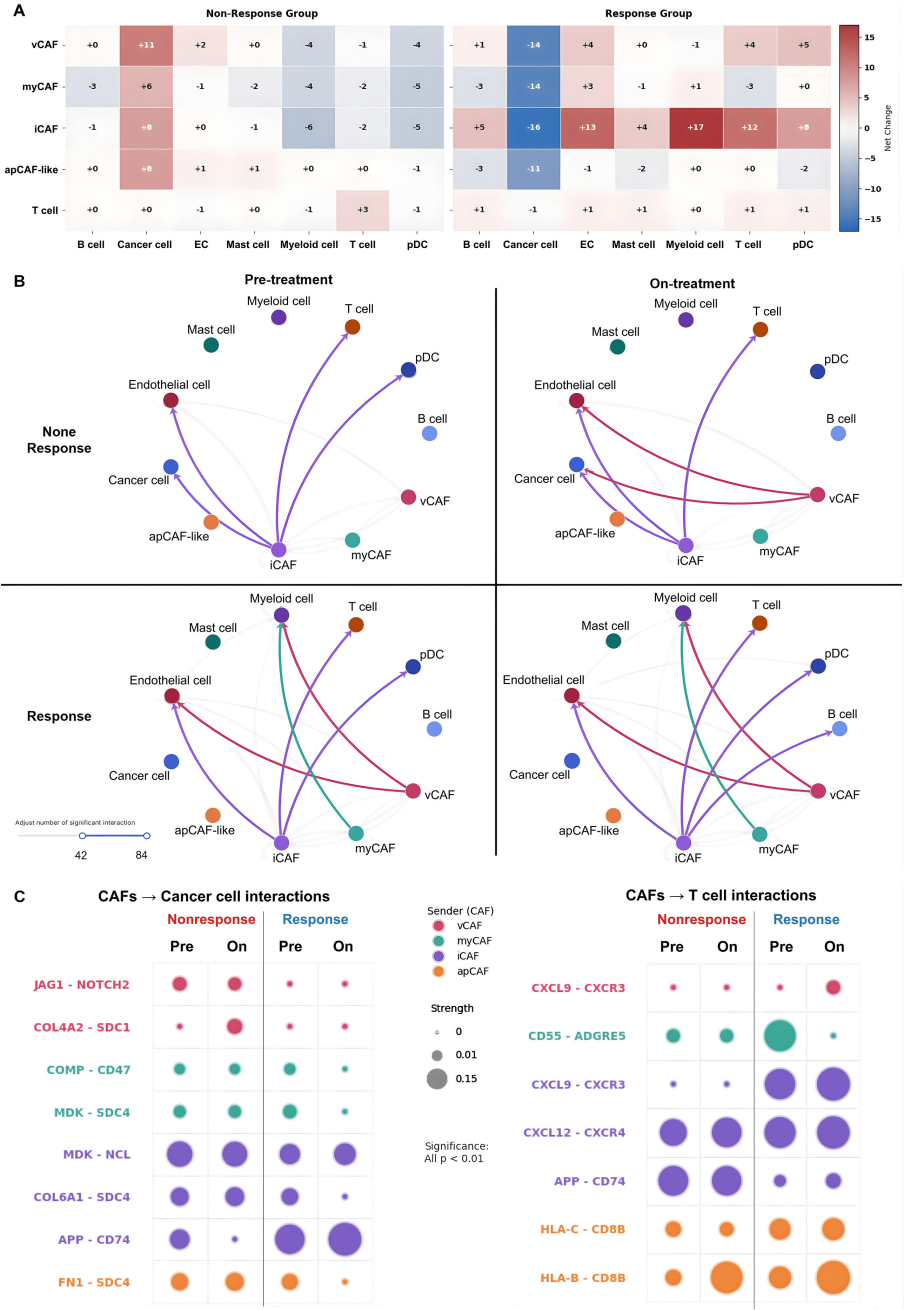
Therapy-induced reprogramming of CAF-mediated intercellular communication networks. **(A)** Global signaling shifts presented as delta values (Δ = *N*_On_ − *N*_Pre_) of CAF-derived interactions. Heatmaps stratify patients into Non-Responders (left) and Responders (right), where red and blue indicate pathway expansion and contraction, respectively. **(B)** Quantification of significant CAF-mediated interactions (≥ 42), highlighting network contraction in non-responders versus selective reconfiguration in responders. **(C)** Ligand-receptor specificity (*P <* 0.01) of CAF subtypes interacting with Cancer cells (left) and T cells (right), where Pre and On denote Pre-treatment and On-treatment stages. Bubble plots depict interaction strength (size) and source subtype (color). EC, endothelial cells; pDC, plasmacytoid dendritic cells.

To decode the cellular determinants governing therapeutic efficacy, we quantified the global landscape of intercellular communication within the tumor microenvironment, revealing a profound bifurcation in interaction trajectories contingent upon treatment outcomes (**Figure 4A**; **Supplementary Data 4**). In non-responders, disease progression was characterized by Stromal Fortification, where vCAFs and iCAFs intensified direct supportive signaling to cancer cells, effectively shielding the tumor niche. In sharp contrast, responders exhibited a distinctive Stromal Remodeling, marked by the synchronous attenuation of tumor-supportive interactions. Notably, while CAF lineages underwent extensive reconfiguration—specifically with iCAFs redirecting signals toward T cells and myeloid cells—the intrinsic interaction repertoire of T cells remained remarkably stable (Δ ≈ 0 to −1). This signaling stasis in the T-cell compartment serves as a critical baseline, demonstrating that the observed therapeutic shift is not driven by an autonomous expansion of immune signaling repertoires, but is instead orchestrated by CAFs actively rewriting the intercellular script. Analysis of high-intensity interaction networks (≥ 42 interactions; **Figure 4B**) corroborated that the CAF signaling hierarchy in responders shifted decisively from a tumor-supporting to an immune-promoting profile.

To elucidate the molecular mechanisms driving these macroscopic shifts, we interrogated specific ligand–receptor pairs (**Figure 4C**; **Supplementary Data 5**), identifying a sophisticated two-pronged reprogramming strategy induced by anti-PD-1 therapy. First, the phenotypic re-education of the iCAF lineage. Analysis of pre-treatment baselines revealed that iCAFs initially engaged T cells via the Amyloid Precursor Protein (APP)–CD74 axis. In responders, this specific iCAF-derived signal was significantly attenuated, replaced by the effective activation of the CXCL9–CXCR3 axis. This molecular switch transforms iCAFs from a physical barrier into an immune recruitment hub, facilitating the infiltration of CD8^+^ and Th1 T cells. Crucially, while iCAF-derived APP signaling diminishes, the APP pathway itself is not extinguished but rather functionally reallocated to distinct stromal subsets (vCAF and apCAF) to sustain antigen presentation, as elucidated in the pathway analysis (Section 3.5). Second, the functional disarmament of protective vCAF and myCAF populations.

In non-responders, vCAFs strongly expressed signals associated with cancer stemness (JAG1–NOTCH2) and basement membrane reinforcement (COL4A2–SDC1). In responders, this specific malignant signalling axis targeting cancer cells was effectively dismantled, thereby removing key survival inputs. Similarly, myCAFs in responders displayed an attenuation of chemical defense mechanisms; interactions involving the complement-regulatory protein CD55 (CD55–ADGRE5) and the anti-phagocytic signal (COMP–CD47) were significantly diminished. Furthermore, apCAF-like cells in the responder group exhibited upregulated MHC Class I molecules (HLA-B/C), directly contributing to antigen presentation. Collectively, these data demonstrate that effective anti-PD-1 therapy necessitates a coordinated disruption of stromal defense systems alongside immune recruitment.

### 3.4 Subtype-specific signaling dynamics: from matrix reinforcement to immune engagement and the context-dependent role of CXCL12

**Figure 5:**
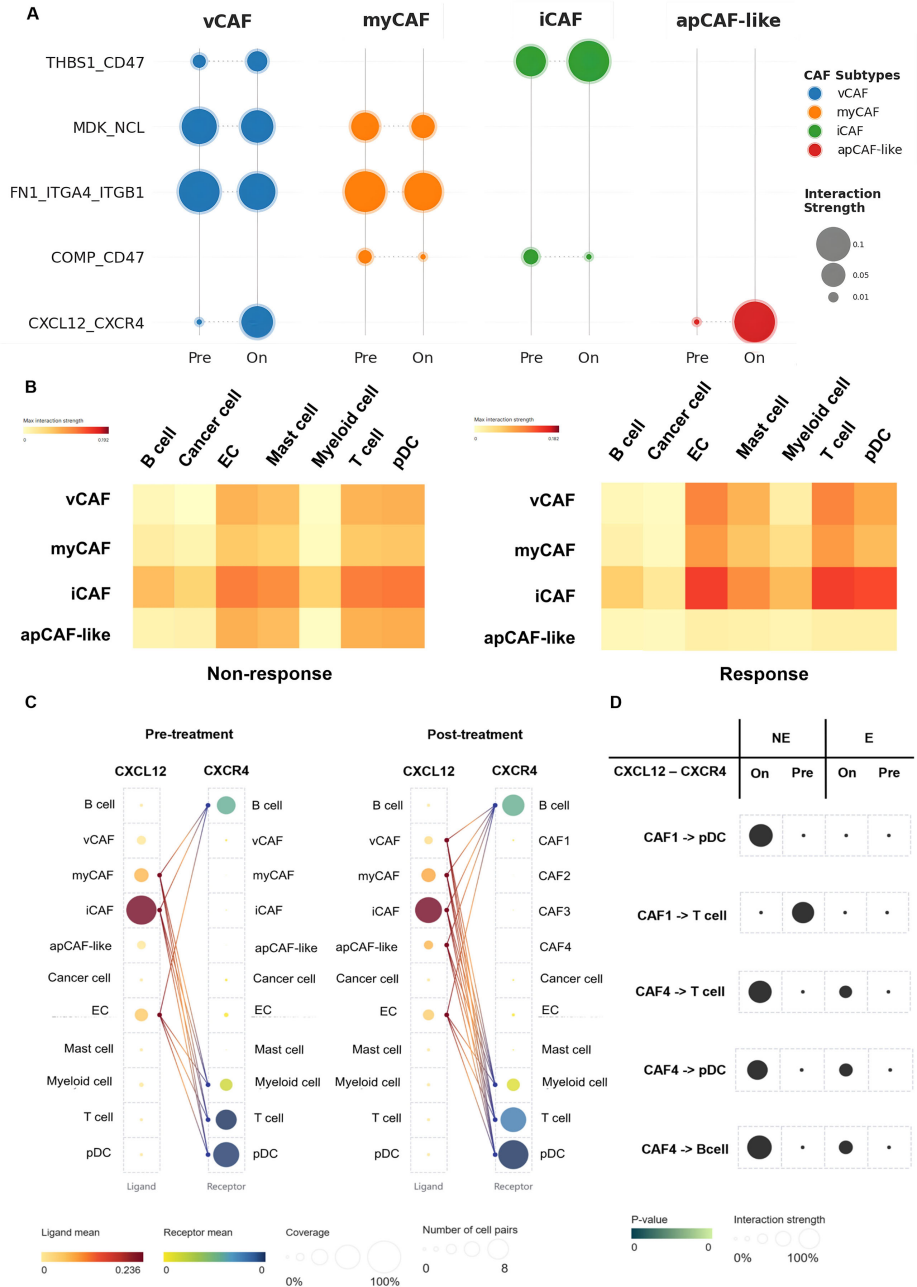
Remodeling of CAF–immune ligand–receptor signaling during anti-PD1 therapy. **(A)** Overview of ligand–receptor pathways that exhibit notable modulation between pre-treatment (Pre) and on-treatment (On) states, filtered for CAF-associated interactions. **(B)** Heatmap quantification of maximum interaction strength for key altered ligand–receptor pathways across clinical conditions (Non-Responders vs. Responders). **(C)** Detailed interaction map of the CXCL12–CXCR4 axis across pre- and post-treatment conditions, showing shifts in ligand/receptor expression and pairwise connections. **(D)** Subtype- and response-specific remodeling of CXCL12–CXCR4 signaling from CAFs to immune cell partners (pDCs, T cells, and B cells), stratified by responder **(E)** versus non-responder (NE) status. EC, endothelial cells; pDC, plasmacytoid dendritic cells.

The detailed ligand-receptor mapping of CAF-mediated cell–cell interactions and signaling dynamics (**Figures 5A, B**; **Supplementary Data 6**) unveils subtype-specific reprogramming with distinct functional consequences. In the case of vCAF, reprogramming is characterized by the upregulation of the Thrombospondin-1–CD47 (THBS1–CD47) signaling pathways. These alterations translate into moderate-strength interactions with endothelial and myeloid populations within the responder group (**Figure 5B**). This enhancement suggests a functional shift toward immune-modulatory activity and immune cell recruitment (38, 39). Concurrently, tumor-promoting signaling axes, such as Midkine–Nucleolin (MDK–NCL) and Fibronectin 1–Integrin (FN1–integrin), are downregulated, which indicates a reduction in ECM-mediated support for tumor growth (40, 41).

In contrast, myCAF exhibits selective functional remodeling rather than a universal attenuation of activity. The downregulation of structural signals like MDK–NCL and COMP–CD47 (**Figure 5A**) suggests a dismantling of the physical barriers that typically exclude immune cells from the tumor microenvironment (42). Crucially, myCAFs within responders maintain strong, high-affinity interactions with T cells and endothelial cells (**Figure 5B**). This stark contrast suggests a phenotypic switch from a barrier-forming phenotype to an immune-permissive state, which actively supports T-cell trafficking and vascular normalization. Similarly, iCAF demonstrates a clear shift towards immune-facing signaling. iCAFs emerge as a central hub of communication in responders, marked by peak interaction strengths with myeloid cells and T cells (**Figure 5B**). This shift is driven by heightened THBS1–CD47 signaling and a reduction in integrin-mediated support, suggesting that iCAFs orchestrate a permissive microenvironment that facilitates robust immune engagement.

The CXCL12–CXCR4 axis, however, exhibited a profound functional divergence between pre-treatment and on-treatment stages, characterized by opposing dynamics in vCAF and apCAF-like populations. (**Figures 5C, D**). In non-responders, therapeutic intervention induced a pathological intensification of vCAF-derived signaling toward plasmacytoid dendritic cells, a feature markedly attenuated in responders. Notably, while vCAF-to-T cell communication was elevated at the pre-treatment baseline in the resistance group, anti-PD-1 therapy triggered a secondary, broad-spectrum surge in apCAF-mediated CXCL12 signaling toward T cells, pDCs, and B cells. Although this apCAF-driven axis was detectable across all patients post-treatment, its magnitude was significantly more pronounced in non-responders. These dynamics suggest that in the context of therapeutic failure, the CXCL12–CXCR4 axis does not facilitate productive immune recruitment but instead orchestrates an immunosuppressive niche characterized by pDC sequestration and dysfunctional lymphoid entrapment.

### 3.5 Divergent signaling architectures: responder-specific APP, NOTCH, and midkine modules versus THBS2–CD47 dominance in resistance

To define the molecular determinants underlying these divergent trajectories, we performed a detailed pathway analysis of ligand–receptor pairs exclusive to each response group (**Table 1**). This analysis revealed a stark dichotomy in signaling programs that was strictly compartmentalized by CAF subtype. Specifically, Responders were characterized by the subtype-restricted activation of Amyloid Precursor Protein (APP), NOTCH, and Midkine (MK) pathways, whereas the Non-response group was dominated by the Thrombospondin-2 (THBS2) axis (43–45). This indicates that therapeutic distinctness is driven by precise functional modules within specific CAF populations rather than ubiquitous stromal activation.

**Table 1:** Predicted cell–cell communications mediated by CAF subtypes through key signaling pathways.

Num	Sig.	Response type*	Ligand	Receptor	Predicted interactions
1	APP	Autocrine dominant – Increase in Response group	APP	TNFRSF21	vCAF → AXL–SIGLEC6 DC, vCAF, Langerhans cell iCAF → vCAF apCAF-like → vCAF, pDC
				SORL1	vCAF →*γδ* T cell apCAF-like → Myeloid cell
				CD74	vCAF → vCAF, myCAF, iCAF, apCAF-like, Malignant epithelial cell iCAF → iCAF apCAF-like → B cell, Endothelial cell, Mast cell, Myeloid cell
				TREM2 + TYROBP	apCAF-like → Myeloid cell
2	NOTCH	Paracrine dominant – Increase in Response group	JAG1	NOTCH1	myCAF → Endothelial cell
				NOTCH2	myCAF → myCAF, iCAF, Langerhans cell, Myeloid cell
				NOTCH3	myCAF → vCAF, myCAF, iCAF
				NOTCH4	myCAF → Endothelial cell
3	MK	Autocrine dominant – Increase in Response group	MDK (MK)	LRP1	apCAF-like → apCAF-like
				ITGA4 + ITGB1	apCAF-like → B cell
				SDC1	apCAF-like → B cell
				NCL	apCAF-like → myCAF, Mast cell, Myeloid cell
4	THBS2	Paracrine dominant – Non-response group	THBS2	CD47	apCAF-like → B cell, iCAF, Malignant epithelial cell, Myeloid cell, pDC, T cell
				SDC1	apCAF-like → myCAF, iCAF
				SDC4	apCAF-like → Malignant epithelial cell
				ITGA3 + ITGB1	apCAF-like → vCAF
				CD36	apCAF-like → Endothelial cell, pDC

*The ‘Response Type’ column indicates the signaling context and functional impact of each pathway.

Specifically, in the responder group, the APP pathway operated as a prominent autocrine and paracrine immune-supportive module, predominantly orchestrated by vCAF and apCAF-like populations. Here, vCAF and apCAF-like cells served as primary ligand sources, interacting with CD74 receptors on a diverse range of targets—including B cells, endothelial cells, mast cells, and myeloid cells. Additionally, vCAF-derived APP engaged SORL1 on *γδ* T cells, while apCAF-like-derived APP targeted TREM2+TYROBP on myeloid lineages. This specific connectivity network promotes antigen cross-presentation and fosters an immune-permissive microenvironment. Similarly, NOTCH signaling in responders was identified as a specific stromal-vascular crosstalk axis driven exclusively by myCAF-derived JAG1. This ligand engaged NOTCH1/4 on endothelial cells and NOTCH2/3 on stromal and myeloid subsets, potentially supporting vascular normalization and stromal remodeling without triggering immunosuppression. Furthermore, the MK pathway emerged as a critical lymphoid-niche organizing mechanism, where apCAF-like cells specifically targeted B cells via MDK–ITGA4+ITGB1 and MDK–SDC1 interactions. This mechanism likely favors B-cell recruitment and tertiary lymphoid structure (TLS) formation.

Conversely, the Non-response group was dominated by a unique broad-spectrum suppressive broadcast driven by apCAF-like cells via the THBS2 pathway. Unlike the spatially coordinated signaling in responders, this resistance-associated module involved apCAF-like-derived THBS2 engaging CD47 on a broad spectrum of targets (B cells, iCAFs, malignant cells, myeloid cells, pDCs, and T cells), as well as NCL, SDC1/4, and CD36 on stromal and endothelial compartments. This specific apCAF-like–THBS2–CD47 axis likely contributes to therapeutic resistance by sustaining an immunosuppressive and exclusionary tumor microenvironment. Collectively, the identification of these response-specific ligand–receptor pairs highlights APP, NOTCH, and MK as potential biomarkers for effective anti-PD-1 therapy, while positing the apCAF-driven THBS2–CD47 axis as a critical target to overcome therapeutic resistance.

### 3.6 Spatial validation of myCAF and iCAF identities and their immune-associated niches

**Figure 6:**
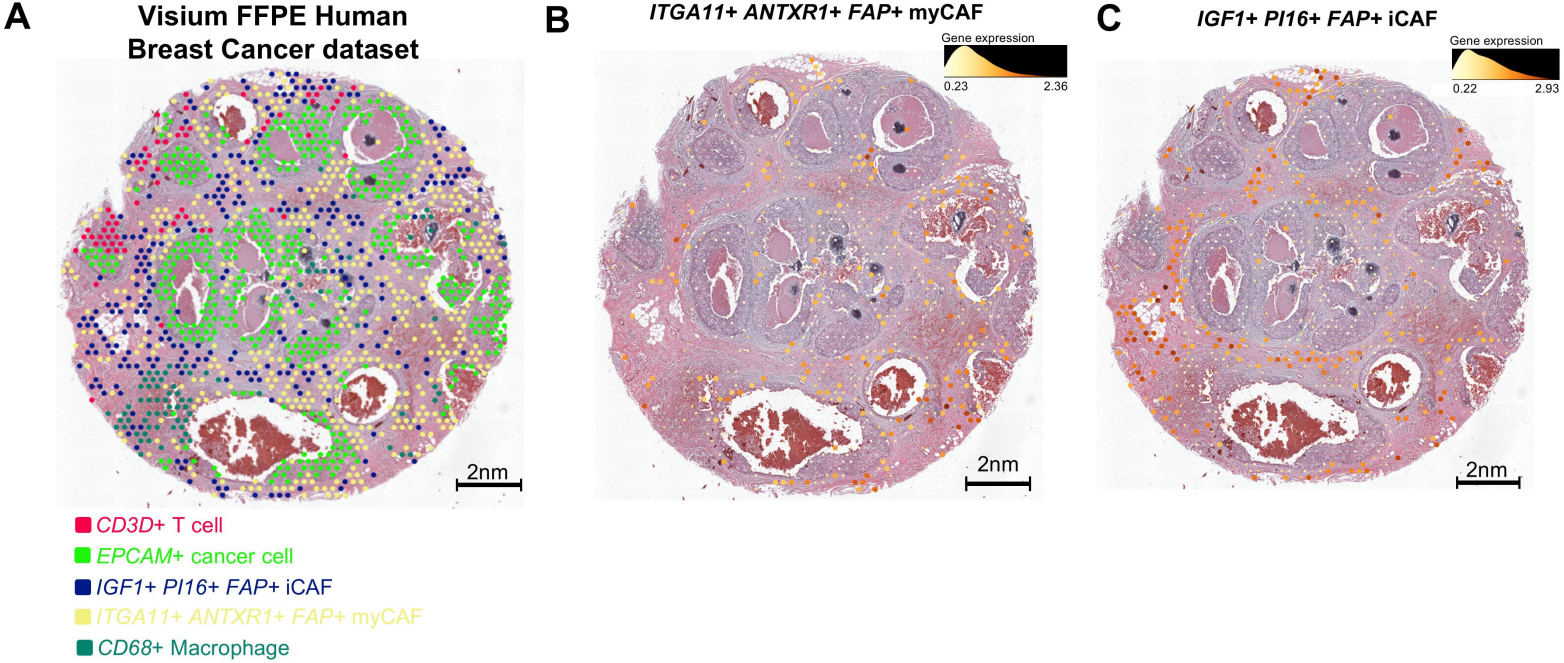
Spatial organization of CAF subtypes is associated with distinct immune contexts. **(A)** Visium spatial transcriptomics map depicting the spatial architecture. **(B)***ITGA11*^+^*ANTXR1*^+^*FAP*^+^myCAF are predominantly enriched in fibrotic stromal regions and display a mutually exclusive distribution with immune cells, consistent with an immune-excluded stromal phenotype. **(C)** Within immune-inflamed stromal areas, *IGF1*^+^*PI16*^+^*FAP*^+^ iCAF spatially associate with immune-rich niches, suggesting potential paracrine interactions within the inflammatory microenvironment. FFPE, Formalin-Fixed Paraffin-Embedded.

To examine the spatial distribution of CAF subtypes within an intact tumor architecture, we analyzed an independent breast cancer spatial transcriptomics dataset (17). Using a two-step spatial mapping strategy, we first assessed whether marker genes derived from our scRNA-seq analysis delineated distinct CAF populations *in situ*, and subsequently evaluated their anatomical localization within the tumor microenvironment. Established reference markers, including *ANTXR1* for myCAFs and *PI16* for iCAFs, together with the pan-fibroblast marker *FAP* (46), were used to contextualize CAF identity. In parallel, subtype-specific markers identified in our scRNA-seq analysis, *ITGA11* for myCAFs and *IGF1* for iCAFs (**Figure 2A**), were spatially mapped alongside these reference markers (**Figure 6A**). This approach delineated two spatially separable CAF populations, defined as *ITGA11*^+^*ANTXR1*^+^*FAP*^+^ myCAFs and *IGF1*^+^*PI16*^+^*FAP*^+^ iCAFs, consistent with their transcriptomic identities.

We next examined the spatial neighborhoods associated with each CAF subtype. *ITGA11*^+^*ANTXR1*^+^ myCAFs were predominantly localized within fibrotic stromal regions and were spatially segregated from immune cell–enriched areas (**Figure 6B**). This spatial distribution is consistent with an immune-excluded stromal architecture and suggests a structural association between myCAF-enriched stroma and limited immune cell accessibility (26, 47). In contrast, the *IGF1*^+^*PI16*^+^ iCAF population exhibited a distinct spatial organization, preferentially localizing to immune-associated regions of the tumor microenvironment (**Figure 6C**). iCAFs were frequently observed in proximity to macrophages and T cells, indicating a baseline anatomical configuration permissive for close stromal–immune spatial association (31, 48). Although spatial transcriptomics captures a static snapshot, this organization provides the spatial context required for the subtype-specific ligand–receptor interactions identified in our network analyses.

## 4 Discussion

Our study has expanded the understanding of the role of cancer-associated fibroblasts (CAFs) in modulating the response to anti-PD-1 therapy. The context of this research arises from previous works, which primarily focused on immune cells and unfortunately overlooked the role of stromal cells. By filling this gap and focusing on the heterogeneity of CAFs, we have identified key subsets that play a crucial role in creating an immunosuppressive tumor microenvironment. These findings reinforce recent evidence, suggesting that the diversity of the tumor stroma is a critical determinant of treatment efficacy, especially in immunotherapy.

In this context, the CAF clusters identified here show strong correspondence with previously defined fibroblast phenotypes across diverse tumor types. This correspondence is supported by cross-dataset validation using shared upregulated genes with published datasets, highlighting the robustness and cross-cancer generalizability of these CAF subtypes (**Supplementary Table S1**). vCAFs, as reflected by the expression of Protein Phosphatase 1 Regulatory Inhibitor Subunit 14A (*PPP1R14A*), Regulator Of G Protein Signaling 5 (*RGS5*), *HIGD1B*, and *MCAM*, exhibited profiles consistent with vascular programs reported in ovarian and breast cancer as well as in normal heart tissue (49, 50). Notably, overlap with *NOTCH3*, Collagen Type XVIII Alpha 1 Chain (*COL18A1*), and Myosin Heavy Chain 11 (*MYH11*) further confirmed its vascular identity while distinguishing it from pericytes, in line with the vCAF cluster described (51). myCAFs, sharing Collagen Type X Alpha 1 Chain (*COL10A1*), Collagen Type XI Alpha 1 Chain (*COL11A1*), Thrombospondin 2 (*THBS2*), Syndecan 1 (*SDC1*), and Podocan Like 1 (*PODNL1*) with fibroblast clusters reported in breast, pancreatic, and colorectal tumors, displayed hallmark collagen- and ECM-remodeling signatures (51–54). iCAFs, characterized by *CXCL12*, Phospholipase A2 Group IIA (*PLA2G2A*), Scavenger Receptor Class A Member 5 (*SCARA5*), and *CFD*, showed consistency with iCAF signatures across breast, thyroid, and colorectal cancers, supporting their role in immune modulation and paracrine signaling (48, 51, 55). By contrast, apCAFs, sharing *CD74* and *HLA-DPA1* but also displaying epithelial and immune admixture signatures, suggest that this population represents a hybrid or context-dependent state rather than a canonical CAF lineage (56–59).

A primary finding of this study is the association of vCAFs with resistance to anti-PD-1 therapy through a mechanism of stromal fortification. In non-responders, disease progression is characterized by intensified supportive signaling from vCAFs and iCAFs to malignant cells, effectively establishing a protective niche. Mechanistically, our intercellular communication analysis identifies the CXCL12–CXCR4 axis as a primary driver of this resistance, predicated on highly context-dependent target specificity. In non-responding patients, therapeutic intervention triggers a pathological intensification of vCAF-derived signaling specifically toward plasmacytoid dendritic cells. This phenomenon suggests a stromal-pDC trap that reinforces immune evasion, potentially by impairing IFN-*α* production and fostering a tolerogenic environment (47, 60). Notably, while apCAF-mediated CXCL12 signaling toward lymphoid subsets increased post-treatment across the entire cohort, its magnitude was significantly more pronounced in the non-response group. This suggests that excessive CXCL12 may orchestrate dysfunctional immune cell sequestration rather than productive recruitment. These observations provide a compelling rationale for utilizing CXCR4 blockade to dismantle these exclusionary barriers (61).

In sharp contrast, responders exhibit a distinctive stromal dismantling, characterized by the functional disarmament of vCAF and myCAF populations. This process effectively abrogates key survival inputs and immune-evasive signals directed at cancer cells. Specifically, the attenuation of the JAG1–NOTCH2 axis in responders validates recent reports identifying this pathway as a central oncogenic driver in breast cancer, where JAG1 facilitates metastasis and diminishes survival by sustaining tumor stemness (62). Furthermore, the downregulation of the CD55–ADGRE5 axis and concomitant stromal defense mechanisms aligns with emerging evidence that cancer-associated fibroblasts establish an exclusionary shield. Recent studies emphasize that CAF-derived interactions, particularly those involving CD55 and extracellular matrix remodeling, are critical for maintaining an immunosuppressive environment and driving therapeutic resistance (63). By dismantling these specific circuits, responders transition from a state of stromal-mediated protection to an immune-permissive environment, thereby facilitating effective anti-PD-1 activity.

Our study further identifies a two-pronged reprogramming strategy induced by anti-PD-1 therapy. First, the re-education of the iCAF lineage represents a pivotal therapeutic shift, characterized by a molecular switch from the inhibitory APP–CD74 baseline toward the immune-recruiting CXCL9–CXCR3 axis. This transformation effectively converts iCAFs from a physical barrier into an immune recruitment hub, facilitating Th1 and CD8^+^ T-cell infiltration. Such functional plasticity aligns with recent high-resolution dissections demonstrating that iCAF subsets can transition from pro-tumorigenic to immune-supportive states under therapeutic pressure (64). Critically, the emergence of iCAF-derived CXCL9 in responders validates recent findings identifying CXCL9 as a fundamental orchestrator of the T-cell inflamed phenotype and a primary determinant of immunotherapy success in breast cancer (65). By redirecting signaling toward this recruitment axis, iCAFs in responders actively prime the microenvironment, confirming that stromal-mediated CXCL9 production is a prerequisite for effective anti-PD-1 activity. Second, responders leverage subtype-specific modules for microenvironmental normalization, including myCAF-mediated JAG1–NOTCH signaling for vascular normalization (66) and apCAF-driven Midkine signaling supporting tertiary lymphoid structure formation. Conversely, the resistance-associated landscape is dominated by a broad-spectrum suppressive broadcast via the apCAF–THBS2–CD47 axis, which sustains a systemic exclusionary environment (67, 68). Collectively, these findings demonstrate that CAF subsets orchestrate divergent stromal programs based on clinical context, establishing the stromal compartment as a highly regulated gatekeeper of immunotherapy success.

While our study primarily focuses on stromal resistance mechanisms in the context of anti–PD-1 therapy, the potential conservation of CAF-mediated immune barriers across other ICIs, including anti–PD-L1 and anti-CTLA-4, is of clear clinical relevance. We anticipate substantial mechanistic overlap with anti–PD-L1 therapies, as both agents target the same inhibitory axis and act predominantly during the effector phase within the tumor microenvironment. Notably, this is the compartment in which vCAFs and apCAF-like populations exert immunosuppressive functions through CXCL12–CXCR4 and THBS2–CD47 signaling, respectively, thereby reinforcing immune exclusion. In contrast, anti-CTLA-4 therapy primarily enhances T-cell priming in secondary lymphoid organs. In this setting, CAFs are likely to function as a downstream resistance bottleneck (60). Even if CTLA-4 blockade effectively expands the peripheral T-cell repertoire, vCAF-mediated angiogenic remodeling and CAF-associated immunosuppressive signaling may still impede effector T-cell infiltration and function within the tumor bed. This concept is consistent with prior reports demonstrating that TGF-*β*–driven stromal programs attenuate therapeutic responses to both anti–PD-L1 and anti-CTLA-4 agents (69–71). Collectively, these observations suggest that targeting specific CAF subtypes—particularly through disruption of stromal TGF-*β* or CXCL12 signaling—may represent a rational combinatorial strategy to overcome resistance across multiple ICI modalities.

This study highlights the central role of the stromal compartment in shaping tumor–immune organization with potential relevance to immunotherapy. Through the integration of stringent quality control, unsupervised clustering, pathway enrichment, and intercellular communication analyses, we provide a high-resolution framework describing CAF heterogeneity and its association with immune architecture. Moving beyond an immune-centric perspective, our findings position stromal–immune crosstalk as an important dimension contributing to therapeutic sensitivity and resistance. Several considerations merit discussion. The overall sample size and the clinical diversity of the cohort, particularly across breast cancer subtypes, may influence the extent to which these observations can be generalized. Notably, the limited representation of HER2-positive tumors constrains subtype-resolved analyses. While the CAF programs identified here may reflect conserved principles of stromal organization and immune modulation, comprehensive pan-cancer validation will require future studies incorporating larger, clinically stratified datasets. With respect to spatial analyses, the available spatial transcriptomics data enabled assessment of the anatomical distribution of major CAF subtypes and their immune-associated niches. However, evaluation of treatment-dependent remodeling and response-linked spatial dynamics will necessitate spatial datasets explicitly annotated with therapeutic exposure and clinical outcome. In addition, the current spatial data provide spot-level representations of tissue architecture rather than true single-cell resolution, limiting the ability to resolve transitional CAF states and dynamic phenotypic plasticity. Finally, as our analyses are primarily transcriptome-based, they do not directly capture post-transcriptional regulation, proteomic dynamics, or the long-term functional stability of CAF programs, which will be important areas for future investigation.

Future studies incorporating longitudinal sampling and multi-omics spatial profiling, including high-resolution proteogenomic approaches, will be essential to clarify CAF plasticity and establish causal relationships. Functional perturbation models, such as organoid co-cultures or *in vivo* systems, will further be required to determine whether specific signaling programs—such as the apCAF-associated THBS2–CD47 axis—directly contribute to immune modulation. Together, our results position CAF heterogeneity as a key stromal dimension associated with immunotherapy response and highlight context-specific CAF signaling pathways as potential candidates for rational combination strategies in breast cancer and other solid tumors.

## Data Availability

Processed single-cell RNA-seq data generated and analyzed in this study are available in Figshare: Pham et al. (2025), Processed single-cell RNA-seq dataset of cancer-associated fibroblasts in breast cancer patients receiving anti–PD-1 therapy (https://doi.org/10.6084/m9.figshare.30663536.v1). This study reanalyzes raw data from the original BioKey study. Raw sequencing reads (scRNA-seq, scTCR-seq, CITE-seq, exome, and low-coverage WGS) are available under controlled access at the European Genome-phenome Archive (EGA) (study no. EGAS00001004809, accession EGAD00001006608). Public read count matrices are accessible at https://lambrechtslab.sites.vib.be/en. Public spatial transcriptomics data used for validation were obtained from 10x Genomics Visium platform: Human Breast Cancer: Ductal Carcinoma In Situ, Invasive Carcinoma (FFPE).

## References

[1] Dvir K Giordano S Leone JP . Immunotherapy in breast cancer. Int J Mol Sci. (2024) 25:7517. doi: 10.3390/ijms25147517, PMID: 39062758 PMC11276856

[2] World Health Organization . Breast cancer – fact sheet (2025). Available online at: https://www.who.int/news-room/fact-sheets/detail/breast-cancer (Accessed November 12, 2025).

[3] Kundu M Butti R Panda VK Malhotra D Das S Mitra T . Modulation of the tumor microenvironment and mechanism of immunotherapy-based drug resistance in breast cancer. Mol Cancer. (2024) 23:92. doi: 10.1186/s12943-024-01990-4, PMID: 38715072 PMC11075356

[4] Biffi G Tuveson DA . Diversity and biology of cancer-associated fibroblasts. Physiol Rev. (2020) 101:147–76. doi: 10.1152/physrev.00048.2019, PMID: 32466724 PMC7864232

[5] Prakash J Shaked Y . The interplay between extracellular matrix remodeling and cancer therapeutics. Cancer Discov. (2024) 14:1375–88. doi: 10.1158/2159-8290.CD-24-0002, PMID: 39091205 PMC11294818

[6] Kieffer Y Hocine HR Gentric G Pelon F Bernard C Bourachot B . Single-cell analysis reveals fibroblast clusters linked to immunotherapy resistance in cancer. Cancer Discov. (2020) 10:1330–51. doi: 10.1158/2159-8290.CD-19-1384, PMID: 32434947

[7] Yang J Xu Q Lu Y . Decoding epithelial–fibroblast interactions in lung adenocarcinoma through single-cell and spatial transcriptomics. J Cancer Res Clin Oncol. (2025) 151:1–12. doi: 10.1007/s00432-025-06250-6, PMID: 40705084 PMC12290149

[8] Emens LA . Breast cancer immunotherapy: facts and hopes. Clin Cancer Res. (2018) 24:511–20. doi: 10.1158/1078-0432.CCR-16-3001, PMID: 28801472 PMC5796849

[9] Bassez A Vos H Van Dyck L Floris G Arijs I Desmedt C . A single-cell map of intratumoral changes during anti-pd1 treatment of patients with breast cancer. Nat Med. (2021) 27:820–32. doi: 10.1038/s41591-021-01323-8, PMID: 33958794

[10] BioTuring Inc . Biovinci: A versatile data visualization platform for life scientists (2025). Available online at: https://app.bioturing.com/xplot (Accessed November 12, 2025).

[11] BioTuring Inc . Bbrowserx: Single-cell analysis platform (2025). Available online at: https://app.bioturing.com/bbrowserx/ (Accessed November 12, 2025).

[12] Ma’ayan Laboratory . Enrichr – interactive gene list enrichment analysis tool (2025). Available online at: https://maayanlab.cloud/Enrichr/ (Accessed November 11, 2025).

[13] Reactome Consortium . Reactome – pathway database of human biological processes (2025). Available online at: https://reactome.org/ (Accessed November 11, 2025).

[14] WikiPathways Consortium . Wikipathways – community-curated biological pathway database (2025). Available online at: https://www.wikipathways.org/ (Accessed November 11, 2025).

[15] Gene Ontology Consortium . Gene ontology – a resource for gene product annotation (2025). Available online at: https://geneontology.org/ (Accessed November 11, 2025).

[16] CellPhoneDB Consortium . Cellphonedb – interactive database for ligand-receptor mediated cell-cell communication (2025). Available online at: https://www.cellphonedb.org/ (Accessed November 12, 2025).

[17] 10x Genomics . Human breast cancer: Ductal carcinoma in *situ*, invasive carcinoma (FFPE) (2021). Available online at: https://www.10xgenomics.com/datasets/human-breast-cancer-ductal-carcinoma-in-situ-invasive-carcinoma-ffpe-1-standard-1-3-0 (Accessed December 18, 2025)

[18] BioTuring Inc . Talk2data: Large-scale single-cell data platform (2025). Available online at: https://app.bioturing.com/talk2data/ (Accessed November 12, 2025).

[19] BioTuring Inc . Spatialx: High-performance spatial transcriptomics analysis platform (2025). Available online at: https://bioturing.com/spatialx (Accessed December 19, 2025).

[20] Kayamori K Katsube KI Sakamoto K Ohyama Y Hirai H Yukimori A . Notch3 is induced in cancer-associated fibroblasts and promotes angiogenesis in oral squamous cell carcinoma. PloS One. (2016) 11:e0154112. doi: 10.1371/journal.pone.0154112, PMID: 27124156 PMC4849776

[21] Brechbuhl HM Barrett AS Kopin E Hagen JC Han AL Gillen AE . Fibroblast subtypes define a metastatic matrisome in breast cancer. JCI Insight. (2020) 5:e130751. doi: 10.1172/jci.insight.130751, PMID: 32045383 PMC7101155

[22] Imaoka T Okutani T Daino K Iizuka D Nishimura M Shimada Y . Overexpression of notch-regulated ankyrin repeat protein is associated with breast cancer cell proliferation. Anticancer Res. (2014) 34:2165–71., PMID: 24778018

[23] Ye QW Liu YJ Li JQ Han M Bian ZR Chen TY . Gja4 expressed on cancer associated fibroblasts (cafs)—a ‘promoter’of the mesenchymal phenotype. Trans Oncol. (2024) 46:102009. doi: 10.1016/j.tranon.2024.102009, PMID: 38833783 PMC11190749

[24] Zhu H Guo S Zhang Y Yin J Yin W Tao S . Proton-sensing gpcr-yap signalling promotes cancer-associated fibroblast activation of mesenchymal stem cells. Int J Biol Sci. (2016) 12:389. doi: 10.7150/ijbs.13688, PMID: 27019624 PMC4807159

[25] Forsthuber A Aschenbrenner B Korosec A Jacob T Annusver K Krajic N . Cancer-associated fibroblast subtypes modulate the tumor-immune microenvironment and are associated with skin cancer Malignancy. Nat Commun. (2024) 15:9678. doi: 10.1038/s41467-024-53908-9, PMID: 39516494 PMC11549091

[26] Iwai M Tulafu M Togo S Kawaji H Kadoya K Namba Y . Cancer-associated fibroblast migration in non-small cell lung cancers is modulated by increased integrin *α*11 expression. Mol Oncol. (2021) 15:1507–27. doi: 10.1002/1878-0261.12937, PMID: 33682233 PMC8096795

[27] Al-Mnaseer ZAM . Investigation into the role of the long non-coding RNAs NEAT1 and MIAT in breast cancer. Ph.D. thesis Keele Univ. (2018).

[28] Cain S Mularczyk E Singh M Massam-Wu T Kielty C . Adamts-10 and-6 differentially regulate cell-cell junctions and focal adhesions. Sci Rep. (2016) 6:35956. doi: 10.1038/srep35956, PMID: 27779234 PMC5078793

[29] Davidson S Coles M Thomas T Kollias G Ludewig B Turley S . Fibroblasts as immune regulators in infection, inflammation and cancer. Nat Rev Immunol. (2021) 21:704–17. doi: 10.1038/s41577-021-00540-z, PMID: 33911232

[30] Joo EH Kim S Park D Lee T Park WY Han KY . Migratory tumor cells cooperate with cancer associated fibroblasts in hormone receptor-positive and her2-negative breast cancer. Int J Mol Sci. (2024) 25:5876. doi: 10.3390/ijms25115876, PMID: 38892065 PMC11172245

[31] Daubriac J Han S Grahovac J Smith E Hosein A Buchanan M . The crosstalk between breast carcinoma-associated fibroblasts and cancer cells promotes rhoa-dependent invasion via igf-1 and pai-1. Oncotarget. (2017) 9:10375. doi: 10.18632/oncotarget.23735, PMID: 29535813 PMC5828213

[32] Yu Z Liu H Ye J Liu Y Xin L Liu Q . Integrative analysis identifies cancer cell-intrinsic rarres1 as a predictor of prognosis and immune response in triple-negative breast cancer. Front Genet. (2024) 15:1360507. doi: 10.3389/fgene.2024.1360507, PMID: 38533207 PMC10963550

[33] Wang H Yn Z Zhang S Liu K Huang R Li Z . Transcriptome-wide analysis reveals potential roles of cfd and angptl4 in fibroblasts regulating b cell lineage for extracellular matrix-driven clustering and novel avenues for immunotherapy in breast cancer. Mol Med. (2025) 31:179. doi: 10.1186/s10020-025-01237-y, PMID: 40340806 PMC12063413

[34] Thomas ME Jie E Kim AM Mayberry TG Cowan BC Luechtefeld HD . Exploring the role of antigen-presenting cancer-associated fibroblasts and cd74 on the pancreatic ductal adenocarcinoma tumor microenvironment. Med Oncol. (2024) 42:15. doi: 10.1007/s12032-024-02564-6, PMID: 39585543

[35] Chen X Chen F Jia S Lu Q Zhao M . Antigen-presenting fibroblasts: emerging players in immune modulation and therapeutic targets. Theranostics. (2025) 15:3332. doi: 10.7150/thno.104900, PMID: 40093895 PMC11905139

[36] Gao ZJ Fang H Sun S Liu SQ Fang Z Liu Z . Single-cell analyses reveal evolution mimicry during the specification of breast cancer subtype. Theranostics. (2024) 14:3104. doi: 10.7150/thno.96163, PMID: 38855191 PMC11155410

[37] Lujano Olazaba O Farrow J Monkkonen T . Fibroblast heterogeneity and functions: insights from single-cell sequencing in wound healing, breast cancer, ovarian cancer and melanoma. Front Genet. (2024) 15:1304853. doi: 10.3389/fgene.2024.1304853, PMID: 38525245 PMC10957653

[38] Ahmed MSU Lord BD Adu Addai B Singhal SK Gardner K Salam AB . Immune profile of exosomes in african american breast cancer patients is mediated by kaiso/thbs1/cd47 signaling. Cancers. (2023) 15:2282. doi: 10.3390/cancers15082282, PMID: 37190208 PMC10136634

[39] Zielińska KA Katanaev VL . The signaling duo cxcl12 and cxcr4: chemokine fuel for breast cancer tumorigenesis. Cancers. (2020) 12:3071. doi: 10.3390/cancers12103071, PMID: 33096815 PMC7590182

[40] Aller EJ Nair HB Vadlamudi RK Viswanadhapalli S . Significance of midkine signaling in women’s cancers: Novel biomarker and therapeutic target. Int J Mol Sci. (2025) 26:4809. doi: 10.3390/ijms26104809, PMID: 40429950 PMC12112249

[41] Chen W Jiang M Zou X Chen Z Shen L Hu J . Fibroblast activation protein (fap)+ cancer-associated fibroblasts induce macrophage m2-like polarization via the fibronectin 1-integrin *α*5*β*1 axis in breast cancer. Oncogene. (2025) 44:2396–412. doi: 10.1038/s41388-025-03359-3, PMID: 40263422

[42] Rock MJ Holden P Horton WA Cohn DH . Cartilage oligomeric matrix protein promotes cell attachment via two independent mechanisms involving cd47 and. αvβ3 integrin. Mol Cell Biochem. (2010) 338:215–24. doi: 10.1007/s11010-009-0355-3, PMID: 20033473 PMC3150962

[43] Lim S Yoo BK Kim HS Gilmore HL Lee Y Hp L . Amyloid-*β* precursor protein promotes cell proliferation and motility of advanced breast cancer. BMC Cancer. (2014) 14:928. doi: 10.1186/1471-2407-14-928, PMID: 25491510 PMC4295427

[44] Pupo M Pisano A Abonante S Maggiolini M Musti AM . Gper activates notch signaling in breast cancer cells and cancer-associated fibroblasts (cafs). Int J Biochem Cell Biol. (2014) 46:56–67. doi: 10.1016/j.biocel.2013.11.011, PMID: 24275097

[45] Cohen S Shachar I . Midkine as a regulator of b cell survival in health and disease. Br J Pharmacol. (2014) 171:888–95. doi: 10.1111/bph.12419, PMID: 24111754 PMC3925027

[46] Croizer H Mhaidly R Kieffer Y Gentric G Djerroudi L Leclere R . Deciphering the spatial landscape and plasticity of immunosuppressive fibroblasts in breast cancer. Nat Commun. (2024) 15:2806. doi: 10.1038/s41467-024-47068-z, PMID: 38561380 PMC10984943

[47] Grout JA Sirven P Leader AM Maskey S Hector E Puisieux I . Spatial positioning and matrix programs of cancer-associated fibroblasts promote t-cell exclusion in human lung tumors. Cancer Discov. (2022) 12:2606–25. doi: 10.1158/2159-8290.CD-21-1714, PMID: 36027053 PMC9633420

[48] Wu SZ Al-Eryani G Roden DL Junankar S Harvey K Andersson A . A single-cell and spatially resolved atlas of human breast cancers. Nat Genet. (2021) 53:1334–47. doi: 10.1038/s41588-021-00911-1, PMID: 34493872 PMC9044823

[49] Loret N Vandamme N De Coninck J Taminau J De Clercq K Blancke G . Distinct transcriptional programs in ascitic and solid cancer cells induce different responses to chemotherapy in high-grade serous ovarian cancer. Mol Cancer Res. (2022) 20:1532–47. doi: 10.1158/1541-7786.MCR-21-0565, PMID: 35749080

[50] Asp M Giacomello S Larsson L Wu C Fürth D Qian X . A spatiotemporal organ-wide gene expression and cell atlas of the developing human heart. Cell. (2019) 179:1647–60. doi: 10.1016/j.cell.2019.11.025, PMID: 31835037

[51] Cords L Tietscher S Anzeneder T Langwieder C Rees M de Souza N . Cancer-associated fibroblast classification in single-cell and spatial proteomics data. Nat Commun. (2023) 14:4294. doi: 10.1038/s41467-023-39762-1, PMID: 37463917 PMC10354071

[52] Wu SZ Roden DL Wang C Holliday H Harvey K Cazet AS . Stromal cell diversity associated with immune evasion in human triple-negative breast cancer. EMBO J. (2020) 39:e104063. doi: 10.15252/embj.2019104063, PMID: 32790115 PMC7527929

[53] Lee HO Hong Y Etlioglu HE Cho YB Pomella V Van den Bosch B . Lineage-dependent gene expression programs influence the immune landscape of colorectal cancer. Nat Genet. (2020) 52:594–603. doi: 10.1038/s41588-020-0636-z, PMID: 32451460

[54] Storrs EP Chati P Usmani A Sloan I Krasnick BA Babbra R . High-dimensional deconstruction of pancreatic cancer identifies tumor microenvironmental and developmental stemness features that predict survival. NPJ Precis Oncol. (2023) 7:105. doi: 10.1038/s41698-023-00455-z, PMID: 37857854 PMC10587349

[55] Lu L Wang JR Henderson YC Bai S Yang J Hu M . Anaplastic transformation in thyroid cancer revealed by single-cell transcriptomics. J Clin Invest 133. (2023) 133:e169653. doi: 10.1172/JCI169653, PMID: 37053016 PMC10231997

[56] Qian J Olbrecht S Boeckx B Vos H Laoui D Etlioglu E . A pan-cancer blueprint of the heterogeneous tumor microenvironment revealed by single-cell profiling. Cell Res. (2020) 30:745–62. doi: 10.1038/s41422-020-0355-0, PMID: 32561858 PMC7608385

[57] Lee JJ Bernard V Semaan A Monberg ME Huang J Stephens BM . Elucidation of tumor-stromal heterogeneity and the ligand-receptor interactome by single-cell transcriptomics in real-world pancreatic cancer biopsies. Clin Cancer Res. (2021) 27:5912–21. doi: 10.1158/1078-0432.CCR-20-3925, PMID: 34426439 PMC8563410

[58] Zilionis R Engblom C Pfirschke C Savova V Zemmour D Saatcioglu HD . Single-cell transcriptomics of human and mouse lung cancers reveals conserved myeloid populations across individuals and species. Immunity. (2019) 50:1317–34. doi: 10.1016/j.immuni.2019.03.009, PMID: 30979687 PMC6620049

[59] Qi Z Liu Y Mints M Mullins R Sample R Law T . Single-cell deconvolution of head and neck squamous cell carcinoma. Cancers. (2021) 13:1230. doi: 10.3390/cancers13061230, PMID: 33799782 PMC7999850

[60] Feig C Jones JO Kraman M Wells RJ Deonarine A Chan DS . Targeting cxcl12 from fap-expressing carcinoma-associated fibroblasts synergizes with anti–pd-l1 immunotherapy in pancreatic cancer. Proc Natl Acad Sci. (2013) 110:20212–7. doi: 10.1073/pnas.1320318110, PMID: 24277834 PMC3864274

[61] Hanley CJ . Thomas GJ. T-cell tumour exclusion and immunotherapy resistance: a role for caf targeting. Br J Cancer. (2020) 123:1353–5. doi: 10.1038/s41416-020-1020-6, PMID: 32830198 PMC7591574

[62] Wang M Yu F Zhang Y Li P . Novel insights into notch signaling in tumor immunity: potential targets for cancer immunotherapy. Front Immunol. (2024) 15:1352484. doi: 10.3389/fimmu.2024.1352484, PMID: 38444855 PMC10912471

[63] Zhang X Zhang X Yang Q Han R Fadhul W Sachdeva A . Comprehensive analysis of adgre5 gene in human tumors: clinical relevance, prognostic implications, and potential for personalized immunotherapy. Heliyon. (2024) 10:e27459. doi: 10.1016/j.heliyon.2024.e27459, PMID: 38501000 PMC10945187

[64] Bonnin E Rodrigo Riestra M Marziali F Mena Osuna R Denizeau J Maurin M . Cd74 supports accumulation and function of regulatory t cells in tumors. Nat Commun. (2024) 15:3749. doi: 10.1038/s41467-024-47981-3, PMID: 38702311 PMC11068745

[65] Hong L Huang F Hu Z Dong Q Kong Y Zheng X . Role of pd-1 in modulating ifn-*γ*-cxcl9/10-cxcr3 signaling in breast cancer. J Biochem Mol Toxicol. (2024) 38:e23842. doi: 10.1002/jbt.23842, PMID: 39588744

[66] Ghosh A Mitra AK . Metastasis and cancer associated fibroblasts: taking it up a notch. Front Cell Dev Biol. (2024) 11:1277076. doi: 10.3389/fcell.2023.1277076, PMID: 38269089 PMC10806909

[67] Liu Z Ba Y Shan D Zhou X Zuo A Zhang Y . Thbs2-producing matrix cafs promote colorectal cancer progression and link to poor prognosis via the cd47-mapk axis. Cell Rep. (2025) 44:115555. doi: 10.1016/j.celrep.2025.115555, PMID: 40222008

[68] Zhao Y Li Y Wang P Zhu M Wang J Xie B . The cancer-associated fibroblasts interact with Malignant t cells in mycosis fungoides and promote the disease progression. Front Immunol. (2025) 15:1474564. doi: 10.3389/fimmu.2024.1474564, PMID: 39963655 PMC11830738

[69] Mariathasan S Turley SJ Nickles D Castiglioni A Yuen K Wang Y . Tgf*β* attenuates tumour response to pd-l1 blockade by contributing to exclusion of t cells. Nature. (2018) 554:544–8. doi: 10.1038/nature25501, PMID: 29443960 PMC6028240

[70] Chakravarthy A Khan L Bensler NP Bose P De Carvalho DD . Tgf-*β*-associated extracellular matrix genes link cancer-associated fibroblasts to immune evasion and immunotherapy failure. Nat Commun. (2018) 9:4692. doi: 10.1038/s41467-018-06654-8, PMID: 30410077 PMC6224529

[71] Tauriello DV Palomo-Ponce S Stork D Berenguer-Llergo A Badia-Ramentol J Iglesias M . Tgf*β* drives immune evasion in genetically reconstituted colon cancer metastasis. Nature. (2018) 554:538–43. doi: 10.1038/nature25492, PMID: 29443964

