# Supplementary material for: Molecular remodeling of cancer-associated fibroblasts in breast cancer patients receiving anti–PD-1 immunotherapy
Source: Front Oncol. 2026 Feb 24;16:1754311. doi: 10.3389/fonc.2026.1754311 (PMC12971403; doi:10.3389/fonc.2026.1754311)
Supplement: Supplementary file 1 [file DataSheet1.pdf]

Supplementary Material

1 Supplementary Data

Supplementary Data 1. Top-ranked CAF marker genes by weighted $\log_2$FC (W$\log_2$FC) and coverage metrics.

This dataset presents the top 100 marker genes for each of the four CAF clusters, prioritized based on weighted $\log_2$FC (W$\log_2$FC, restricted to values > 0). The integration of Within-cluster Coverage, Outside-cluster Coverage, and Log2FC scores was used to rank these genes. The table shows representative high-ranking genes from each CAF cluster (CAF1-CAF4), with information on rank, gene symbol, coverage values, $\log_2$FC, W$\log_2$FC score, and cluster annotation.

Supplementary Data 2. Differential gene expression analysis of CAF subtypes.

This dataset presents the results of a differential gene expression analysis between each CAF subtype and all other stromal cells. The analysis was performed using the MAST (RRID:SCR_016340) model with two-sided testing, and p-values were adjusted for multiple testing using the Bonferroni correction. Genes with $\log_2$FC > 1 and FDR < 0.05 were considered significant. The table includes columns for Gene name, FDR, $\log_2$FC, Group 1 (CAF subtype), Group 2 (Others), raw p-value, and assigned cluster.

Supplementary Data 3. Functional and pathway enrichment analysis of CAF subtypes.

This dataset summarizes the results of functional and pathway enrichment analyses conducted separately for each CAF cluster (CAF1-CAF4) using multiple databases, including Gene Ontology (RRID:SCR_002811), Reactome (RRID:SCR_003485), and WikiPathways (RRID:SCR_002134). Enrichment analyses were performed based on significantly upregulated genes in each CAF subtype. Pathways with both FDR < 0.05 and p-value < 0.05 were considered significantly enriched. Each table includes columns: Term, which indicates the name or description of the enriched functional category or pathway; Matched genes, listing the genes from the input list associated with each pathway; Database, specifying the source database; p-value and FDR; NES (Normalized Enrichment Score) and ES (Enrichment Score); Gene count, showing the number of input genes present in the pathway; Gene percent, the proportion of input genes represented in the pathway relative to the total input list; and Tag percent, the percentage of genes in the pathway that are found in the input list, reflecting the coverage of the pathway by the input genes.

Supplementary Data 4. Quantification of CAF-mediated cell–cell interactions stratified by clinical response and breast cancer subtypes.

This dataset presents the comprehensive landscape of intercellular communication, quantifying the number of significant ligand–receptor interactions ($P < 0.01$ as determined by CellChat (RRID:SCR_021946) permutation tests) between CAF subtypes (vCAF, myCAF, iCAF, apCAF-like) and other microenvironmental components (immune, stromal, and malignant cells). The analysis was conducted using the CellChat (RRID:SCR_021946) framework to infer signaling networks. The data is provided as interaction count matrices, stratified by clinical outcome (Responders vs. Non-responders)

and therapeutic timepoint (Pre-treatment vs. On-treatment). Additionally, to capture subtype-specific signaling dynamics, the results are categorized into three cohorts: the aggregate dataset ("All"), Estrogen Receptor-positive ("ER+ type"), and Triple-Negative Breast Cancer ("TNBC type"). The tables are organized with sender cells in rows and receiver cells in columns, with values indicating the total number of significant interactions identified for each cell-cell pair.

Supplementary Data 5. Detailed interaction strengths of ligand–receptor pairs between CAF subtypes and key target populations.

This dataset provides the specific interaction strengths for ligand–receptor pairs mediated by CAF subtypes (vCAF, myCAF, iCAF, and apCAF-like) targeting malignant epithelial cells, T cells, and myeloid cells. Interaction strengths were calculated using the CellChat (RRID:SCR_021946) framework, stratified by clinical outcome (Responders vs. Non-responders) and treatment timepoints (Pre-treatment vs. On-treatment). Only interactions achieving statistical significance at $p < 0.01$ are included. Each sub-dataset corresponds to a specific sender–receiver interaction axis (e.g., vCAF -> Cancer cell, iCAF -> T cell, iCAF -> Myeloid cell). The tables include detailed columns for the Interaction pair name, the specific Ligand and Receptor involved, the Interaction strength value, and the associated p-value. This granular information supports the identification of specific molecular drivers behind the divergent stromal trajectories observed in therapeutic response and resistance.

Supplementary Data 6. Pairwise interaction dynamics of key ligand–receptor signaling axes across CAF subsets.

This dataset provides a detailed breakdown of cell–cell communication for specific, high-priority ligand–receptor pairs (e.g., THBS1–CD47, CXCL12–CXCR4, APP–CD74, and THBS2–CD47) that define the functional landscape of CAF subtypes during therapy. For each signaling axis, interaction details are stratified by therapeutic timepoints (Pre-treatment vs. On-treatment). The dataset includes all significant communicative links with a threshold of $P < 0.05$. The tables include specific metrics for each pair, including the Sender cell population, the Receiver cell population, the calculated Interaction strength, and the associated p-value. These data illustrate the precise cellular connectivity and molecular remodeling of individual signaling pathways within the tumor microenvironment in response to anti-PD-1 treatment.

2 Supplementary Figures and Tables

2.1 Supplementary Figures

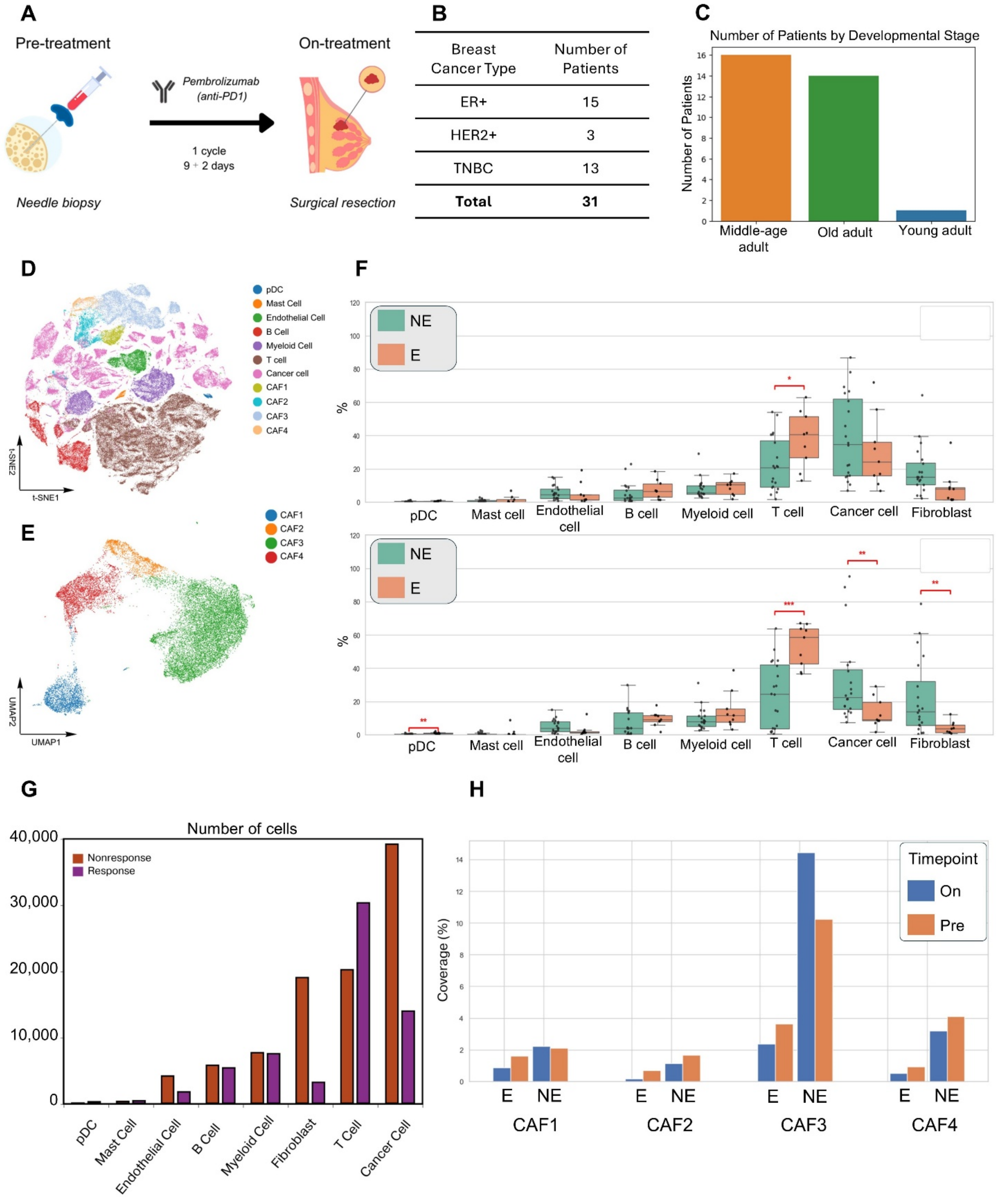

A
Pre-treatment
Pembrolizumab (anti-PD1)
1 cycle
9 ± 2 days
On-treatment
Needle biopsy
Surgical resection
B
Breast Cancer Type | Number of Patients
ER+ | 15
HER2+ | 3
TNBC | 13
Total | 31
C
Number of Patients by Developmental Stage
Number of Patients
Middle-age adult
Old adult
Young adult
D
pDC
Mast Cell
Endothelial Cell
B Cell
Myeloid Cell
T cell
Cancer cell
CAF1
CAF2
CAF3
CAF4
t-SNE1
t-SNE2
E
CAF1
CAF2
CAF3
CAF4
UMAP1
UMAP2
F
NE
E
%
pDC
Mast cell
Endothelial cell
B cell
Myeloid cell
T cell
Cancer cell
Fibroblast
Pre Treatment
On Treatment
G
Number of cells
Nonresponse
Response
40,000
30,000
20,000
10,000
0
pDC
Mast Cell
Endothelial Cell
B Cell
Myeloid Cell
Fibroblast
T Cell
Cancer Cell
H
Coverage (%)
Timepoint
On
Pre
E
NE
CAF1
CAF2
CAF3
CAF4

Supplementary Figure S1. Single-cell profiling of treatment-naive breast cancer patients receiving anti-PD-1 therapy. **(A)** Schematic of the original BioKey single-cell study in treatment-naive breast cancer patients receiving one dose of anti-PD-1, adapted from Bassez et al., Nat Med 2021 (https://pubmed.ncbi.nlm.nih.gov/33958794/) **(B)** Tumor subtype distribution. Table summarizing the number of patients per breast cancer subtype (ER+, HER2+, TNBC), providing an overview of cohort composition for downstream analyses. **(C)** Developmental stage distribution. Bar plot depicting the number of patients in each developmental stage (young adult, middle-age adult, old adult). **(D)** tSNE map of 175,942 cells color-coded for the indicated cell type. **(E)** UMAP representation of CAF subtypes. Four transcriptionally distinct CAF subtypes (CAF1–CAF4) identified via unsupervised clustering, highlighting heterogeneity within the stromal com-partment. **(F)** Relative contribution (in %) of each major cell type at the pre-treatment (upper) and on-treatment (lower) time points, comparing patients with clonotype expansion (E, $n = 9$) versus patients with limited or no clonotype expansion (NE, $n = 20$). **(G)** Cellular composition by treatment response. Bar plot showing the total number of cells for each major cell type, stratified by responders and non-responders, emphasizing differential abundances across lineages. **(H)** CAF abundance differences. Comparison of the total numbers of the four CAF subtypes between non-responders and responders at both pre-treatment and on-treatment stages, demonstrating marked differences in stromal populations prior to detailed subtype-specific analyses. Exact *P* values were calculated by independent t-test for paired samples (pre- versus on-treatment): $*P < 0.05$, $**P < 0.01$, $***P < 0.001$. Boxes indicate median ± interquartile range; whiskers show minima and maxima. pDC, plasmacytoid dendritic cell.

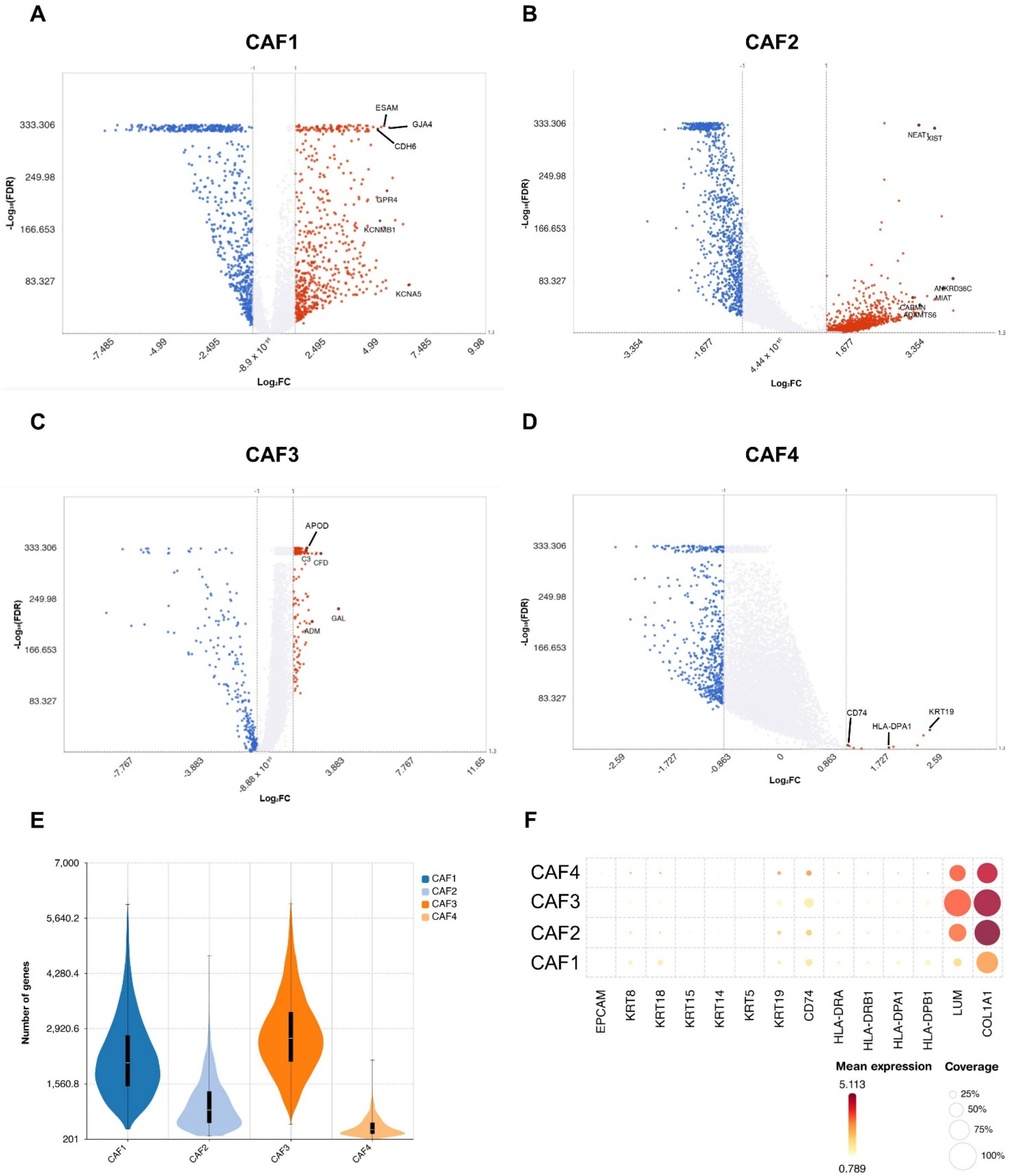
A
CAF1
B
CAF2
C
CAF3
D
CAF4
E
F
ESAM
GJA4
CDH6
GPR4
KCNMB1
KCNA5
NEAT1
XIST
ANKRD36C
MIAT
CARMN
ADAMTS6
APOD
C3
CFD
GAL
ADM
CD74
HLA-DPA1
KRT19
-Log10(FDR)
Log2FC
Number of genes
CAF1
CAF2
CAF3
CAF4
EPCAM
KRT8
KRT18
KRT15
KRT14
KRT5
KRT19
CD74
HLA-DRA
HLA-DRB1
HLA-DPA1
HLA-DPB1
LUM
COL1A1
Mean expression
5.113
0.789
Coverage
25%
50%
75%
100%

Supplementary Figure S2. Transcriptional Characterization and Lineage Validation of CAF Subtypes. **(A–D)** Volcano plots showing differentially expressed genes for each CAF cluster: **(A)** vCAF, **(B)** myCAF, **(C)** iCAF, and **(D)** apCAF-like. Marker genes used to define each subtype are highlighted based on fold change and false discovery rate (FDR) thresholds. **(E)** Violin plot displaying the distribution of detected gene counts across the four clusters to assess potential technical artifacts. **(F)** Bubble heatmap illustrating the expression levels and coverage of lineage-specific markers, including epithelial genes, apCAF-associated MHC-II molecules, and core fibroblast markers (*LUM, COL1A1*), to confirm the bona fide fibroblast identity of the apCAF-like (CAF4) population.

2.2 Supplementary Tables

CAF Subtypes	STUDY ID	AUTHOR LABEL	SHARED MARKERS	TISSUE	CONDITION
vCAF	GSE201047	Vascular cancer-associated fibroblast (vCAF)	*PPP1R14A, RGS5, HIGD1B, COX4I2, MCAM, NDUFA4L2, GJA4, CCDC102B, TPPP3, ANGPT2*	Omentum, urinary bladder, ovary, peritoneum	Ovarian serous adenocarcinoma
	GSE201047	Vascular cancer-associated fibroblast (vCAF)	*PLN, MYH11, RERGL, PPP1R14A, RGS5, COX4I2, GJA4, HIGD1B, MCAM, WFDC1*	Ovary, peritoneum, omentum, urinary bladder	Ovarian serous adenocarcinoma
	PMID31835037	8. Fibroblast-like (related to larger vascular development)	*ENPEP, COX4I2, FOXS1, THBS4, SEPT4, NDUFA4L2, CCDC102B, KCNJ8, HIGD1B, TBX2*	Heart	Normal
	PMID37463917_BC	vCAF	*RERGL, MYH11, BCAM, CASQ2, SORBS2, ACTG2, WFDC1, TINAGL1, PLN, FHL5*	Breast	Breast cancer

CAF Subtypes	STUDY ID	AUTHOR LABEL	SHARED MARKERS	TISSUE	CONDITION
myCAF	SCP1106	Myofibroblast-like cancer-associated fibroblast (myCAFs)	*AEBP1, SFRP2, COL10A1, THBS2, MXRA5, LUM, COL3A1, RARRES2, COL12A1, COL5A1*	Breast	Triple-negative breast carcinoma, invasive breast carcinoma
	GSE176078 _SCP1039	CAFs myCAF like s4	*COL3A1, LUM, COL1A2, COL1A1, POSTN, COL6A3, DCN, COL5A2, COL6A2, THY1*	Breast	Triple-negative/HER2 positive/ estrogen-receptor positive breast carcinoma
	GSE242230	Myofibroblast cancer-associated fibroblast (myCAF)	*COL11A1, COL10A1, THBS2, COL5A1, LUM, SFRP2, COL6A3, COL1A2, AEBP1, COL5A2*	Pancreas, body of pancreas, head of pancreas, neck of pancreas	Pancreatic ductal adenocarcinoma
iCAF	GSE176078 _SCP1039	CAFs MSC iCAF-like	*DCN, MFAP4, LUM, CTSK, SFRP2, C1S, CXCL12, MEG3, RARRES2, PTGDS*	Breast	Triple-negative/HER2 positive/ estrogen-receptor positive breast carcinoma
	GSE193581	Inflammatory cancer-associated fibroblast (iCAF)	*SFRP2, LUM, DCN, C1S, C1R, CTSK,*	Thyroid gland	Thyroid gland undifferentiated (anaplastic) carcinoma,

CAF Subtypes	STUDY ID	AUTHOR LABEL	SHARED MARKERS	TISSUE	CONDITION
			FGF7, NNMT, FBLN1		thyroid gland papillary carcinoma
	SCP1106	Inflammatory-like cancer-associated fibroblast (iCAF)	*LUM, MEG3, DCN, FIGF, CXCL12, CTSK, COL3A1, SRPX, RARRES2, COL1A2*	Breast	Her2-receptor negative breast cancer
	PMID33033240	Inflammatory cancer-associated fibroblast (iCAF)	*CLMP, PDPN, PDGFRA, COL8A1, EMILIN1, CTSK, OLFM3, LINC01082, COL6A3, MXRA8*	Urinary bladder, mucosa of urinary bladder	Urothelial carcinoma
apCAF-like	GSE156405 _FIG1A	Antigen-presenting CAF (apCAF)	*COL1A2, COL3A1, SPARC, COL1A1, AEBP1, DCN, COL6A2, EMILIN1, COL5A1, BGN*	Liver, peritoneum, pancreas, lung, vagina	Pancreatic ductal adenocarcinoma

CAF Subtypes	STUDY ID	AUTHOR LABEL	SHARED MARKERS	TISSUE	CONDITION
	PMID32561858_ LC	SERPINE1+ CAF	*COL3A1, COL1A2, COL1A1, DCN, CCDC80, LUM, BGN, COL6A3, COL6A2, MMP2*	Lung	Lung cancer
	GSE234933	Fibro_TWIST2	*COL3A1, COL1A2, COL6A3, COL1A1, DCN, LUM, AEBP1, COL5A2, SFRP2, CTSK*	Subdivision of tube, oral cavity, lung, oropharynx, nasopharyn x, craniocervic al region, skin of body	Head and neck squamous cell carcinoma
	GSE127465 _HUMAN	Fibro4_COL6A1/ COL6A2	*COL3A1, LUM, COL1A2, COL5A1, POSTN, COL6A3, COL5A2, COL6A2, DCN*	Lower lobe of right lung, upper lobe of right lung, upper lobe of left lung	Non-small cell squamous lung carcinoma, non-small cell lung carcinoma

Supplementary Table S1. Cross-dataset validation and comparative annotation of CAF subtypes based on shared upregulated genes (Supplementary Data 2) with published datasets in Bioturing software.
